# Lithium hexamethyldisilazide as electrolyte additive for efficient cycling of high-voltage non-aqueous lithium metal batteries

**DOI:** 10.1038/s41467-022-34717-4

**Published:** 2022-11-15

**Authors:** Danfeng Zhang, Ming Liu, Jiabin Ma, Ke Yang, Zhen Chen, Kaikai Li, Chen Zhang, Yinping Wei, Min Zhou, Peng Wang, Yuanbiao He, Wei Lv, Quan-Hong Yang, Feiyu Kang, Yan-Bing He

**Affiliations:** 1 Shenzhen All-Solid-State Lithium Battery Electrolyte Engineering Research Center, Institute of Materials Research (IMR), Tsinghua Shenzhen International Graduate School, Tsinghua University, Shenzhen, 518055 China; 2 School of Materials Science and Engineering, Tsinghua University, Beijing, 100084 China; 3 School of Materials Science and Engineering, Harbin Institute of Technology, Shenzhen, 518055 China; 4 School of Pharmacy, East China University of Science and Technology, Shanghai, 200237 China; 5 Nanoyang Group, State Key Laboratory of Chemical Engineering, School of Chemical Engineering and Technology, Tianjin University, Tianjin, 300072 China

**Keywords:** Batteries, Energy, Materials for energy and catalysis, Energy storage

## Abstract

High-voltage lithium metal batteries suffer from poor cycling stability caused by the detrimental effect on the cathode of the water moisture present in the non-aqueous liquid electrolyte solution, especially at high operating temperatures (e.g., ≥60 °C). To circumvent this issue, here we report lithium hexamethyldisilazide (LiHMDS) as an electrolyte additive. We demonstrate that the addition of a 0.6 wt% of LiHMDS in a typical fluorine-containing carbonate-based non-aqueous electrolyte solution enables a stable Li||LiNi_0.8_Co_0.1_Mn_0.1_O_2_ (NCM811) coin cell operation up to 1000 or 500 cycles applying a high cut-off cell voltage of 4.5 V in the 25 °C−60 °C temperature range. The LiHMDS acts as a scavenger for hydrofluoric acid and water and facilitates the formation of an (electro)chemical robust cathode|electrolyte interphase (CEI). The LiHMDS-derived CEI prevents the Ni dissolution of NCM811, mitigates the irreversible phase transformation from layered structure to rock-salt phase and suppresses the side reactions with the electrolyte solution.

## Abstract (web summary)

High-voltage non-aqueous lithium metal batteries suffer from poor cycling stability due to the presence of impurities in the electrolyte solution. Here, the authors report lithium hexamethyldisilazide to scavenge HF and H_2_O, prevent the Ni dissolution and suppress side reactions during cycling.

## Introduction

Nickel-rich cathode such as LiNi_0.8_Co_0.1_Mn_0.1_O_2_ (NCM811) show significant promises to help lithium-ion batteries to achieve high specific energy (>400 W h kg^−1^)^1–4^, while many serious issues still remain including severe side reactions with conventional fluorine-containing carbonate-based liquid electrolyte solutions^5–8^, rapid structural degradation^9,10^, thermodynamic instability^11^ and capacity decay^12^ during cycling under high cut-off voltage (i.e., ≥4.5 V vs Li/Li^+^) and high operation temperature (i.e., ≥60 °C). The Ni^4+^ ions of delithiated NCM811 cathode are easily reduced to Ni^2+^ and Ni^3+^ ions by oxidation reaction of electrolyte, which leads to gas generation and impedance increase of the batteries^9^. Cation mixing of Ni^2+^ and Li^+^ also occurs to form rock-salt phase owning to their similar ionic radius, which leads to structure degradation and huge capacity loss, especially at 60 °C^10–13^. It is generally considered that the trace amount of H_2_O in the electrolyte reacts with PF_6_^−^ of the lithium hexafluorophosphate (LiPF_6_) to generate corrosive hydrofluoric acid (HF)^14–16^, which causes transition metal (TM) ions dissolution^17^, oxygen evolution from cathode surface, aluminum collector corrosion and irreversible cathode-electrolyte interphase (CEI) damage upon cycling and storage^11,18^. The decomposition of the LiPF_6_ salt exhibits strong correlation with the battery operation temperature and the thermal aggravation strongly promotes the salt decompositions^19^. In addition, the instable CEI at high cut-off voltage and high temperature causes excessive electrolyte oxidization and interface deterioration. Addressing these crucial issues is of great significance to pursue an effective strategy to achieve high-performance Li||NCM811 batteries at a wide operation temperature and high cut-off voltage.

Many efforts including doping, surface coating and electrolyte additives have been made to improve the structural and interfacial stability of NCM811 particles. For instance, doping of Ca^2+^^19^, Al^3+^^17^, Zr^4+^^20^, and Nb^5+^^21^ can improve the structure stability of NCM811 by enlarging the Li slab distance. Constructing surface coating layers such as Al_2_O_3_^22^, ZrO_2_^23,24^, and V_2_O_5_^25^ on NCM811 particles can suppress the decomposition of electrolyte at early cycling stage to prevent the formation of metal fluorides and dissolution of TM ions induced by HF. However, the metal oxide coating layers easily react with HF, especially at high cut-off voltage and high temperature. Employing electrolyte additives to stabilize the CEI is an expeditious and convenient method to improve the performance of nickel-rich NCM by scavenging the H_2_O, HF and PF_5_ species^26^. For instance, 4-fluorophenyl isocyanate^27^, dimethoxydimethylsilane (DODSi)^28^, and 3-(trimethylsilyl)−2-oxazolidinone (TMS-ON)^17^ could react with HF to suppress the interfacial reactions of cathode and electrolyte. The aluminum isopropoxide (AIP) could initiate in situ polymerization of ethylene carbonate (EC) to construct a polycarbonate-rich CEI on NCM811^29^. However, the operation temperature of nickel-rich NCM batteries using above additives is limited to 45 °C owning to the reactivity of nickel-rich NCM cathode with liquid electrolyte, which causes the battery overcharge at higher temperature^30^. In addition, the CEI built by additives isn’t (electro)chemical robust and stable sufficiently to inhibit the catalytic side reactions between NCM811 cathode and electrolyte^31^. In practical operating conditions, the battery is heated due to the continuous heat generation^32^. Therefore, promoting the thermal stability of NCM811 batteries at higher than 45 °C is in urgent need for practical application. In addition, the onset oxidation potential of these additives on high nickel cathode is larger than 4 V, which is higher than the initial delithiated potential of NCM811 cathode (3.62 V)^28^. The late formed CEI cannot timely suppresses the side reactions of cathode and non-aqueous electrolyte. Therefore, it is quite significant and critical to develop low oxidation potential electrolyte additives with integrated functions to simultaneously scavenge HF and preferentially construct CEI on NCM811 for advanced Li||NCM811 batteries under high voltage and elevated operation temperature.

Herein, we present a new multi-functional additive, lithium hexamethyldisilazide (LiHMDS) with a low oxidation potential, for NCM811, which improve the cycling stability of Li||NCM811 batteries at high-stress conditions such as high voltage (4.5 V) and high temperature (60 °C). The LiHMDS as a strong organic base with low onset oxidation potential not only scavenges HF and H_2_O in electrolyte, but also preferentially creates a thin and robust CEI on NCM811 before oxidization of electrolyte, which effectively suppresses the irreversible structure transformation of NCM811 particles from layered structure to rock-salt phase, the dissolution of transition metal elements, and side reactions with electrolyte during long cycles. As a result, the Li||NCM811 coin cells with only 0.6 wt% LiHMDS in 1 M LiPF_6_ carbonate-based electrolyte solution can stably cycle for 1000 times at 25 °C and 500 times at 60 °C under high cut-off voltage of 4.5 V, also using 50 μm thick Li metal negative electrodes and high mass-loading positive electrodes (i.e., 10 mg cm^−2^) at 60 °C.

## Results

### Electrochemical performance of Li||NCM811 cells with LiHMDS

**Fig. 1:**
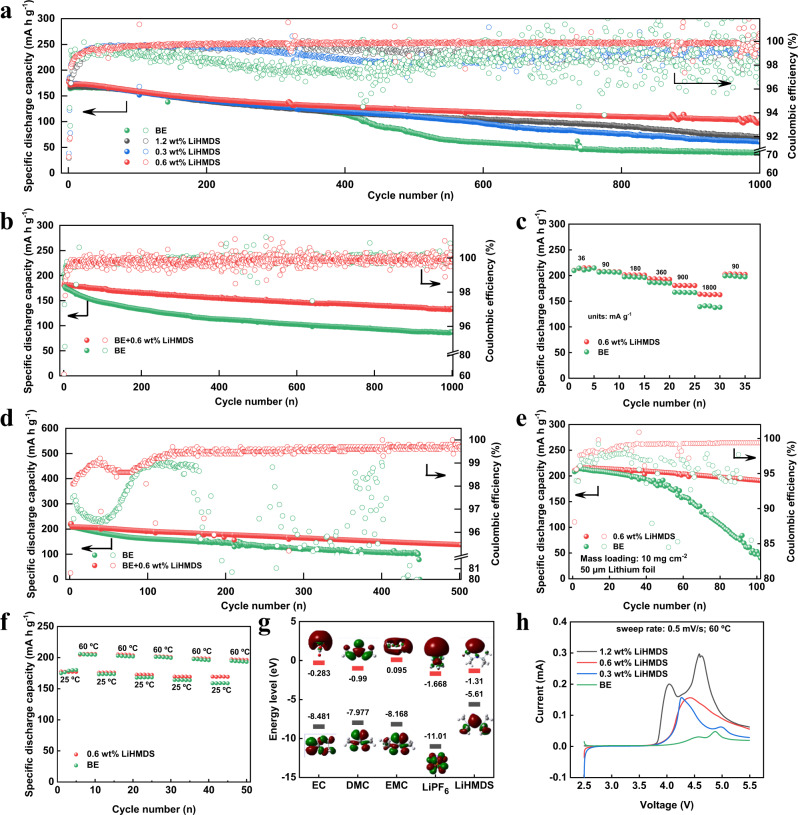
Electrochemical performance of Li||NCM811 cells with and without LiHMDS. **a** Cycling performance of Li||NCM811 cells with different amount of LiHMDS at 25 °C between 2.8 and 4.3 V, specific current is 180 mA g^−1^. **b** Cycling performance at 25 °C between 3 and 4.5 V, specific current is 90 mA g^−1^. **c** Rating performance at 25 °C. **d** Cycling performance at 60 °C between 3 and 4.5 V, specific current is 180 mA g^−1^. **e** Cycling performance of thin Li electrode || high loading NCM811 cells at 60 °C between 3 and 4.5 V, specific current is 60 mA g^−1^. **f** Cycling performance under different temperature between 3 and 4.5 V, specific current is 60 mA g^−1^. **g** HOMO and LUMO energy levels of EC, DMC, EMC, LiPF_6_ and LiHMDS. **h** linear sweep voltammetry measurements of BE and BE with different amount of LiHMDS additives at 60 °C under sweep rate of 0.5 mV s^−1^.

The cycling performance of the Li||NCM811 cells between 2.8 and 4.3 V at 25 °C is improved by LiHMDS as shown in Fig. 1a and Supplementary Fig. 1. The Li||NCM811 cell with 0.6 wt% LiHMDS (0.6 wt% LiHMDS was applied in the following cells with LiHMDS) presents the highest capacity retention of 71.24% after 1000 cycles at 90 mA g^−1^, which is higher than that of Li||NCM811 cells (only using baseline electrolyte (BE) without LiHMDS) (27.12%). The coin cell was dissembled and noticed that a large area of cycled NCM811 cathode of BE cells spalled from the collector after ~400 cycles (Supplementary Fig. 2a). In addition, it is also possible to notice TMs dissolution, which transferred to lithium metal anode (LMA) (Supplementary Fig. 2b). In sharp contrast, the cycled NCM811 cathode from LiHMDS cells presents intact structure after 400 cycles and the dissolution of transition metals seems to be hindered. Therefore, we speculate that the spalling of NCM811 cathode from the collector and dissolution of transition metals result in rapid capacity decline of BE cells after 400 cycles (Supplementary Fig. 2c and d). The cyclic voltammetry (CV) curves show that the Li||NCM811 cells with LiHMDS present better reversibility than the Li||NCM811 cells during 5 cycles from 3 to 4.5 V at both 25 and 60 °C (Supplementary Fig. 3). Accordingly, the Li||NCM811 cells with LiHMDS show higher capacity retention of 73.92% than Li||NCM811 cells (49.13%) after 1000 cycles between 3 and 4.5 V at 90 mA g^−1^ and 25 °C (Fig. 1b). In addition, the Li||NCM811 cells with LiHMDS exhibit better rate performance than the Li||NCM811 cells at 25 ± 1 °C (Fig. 1c). More significantly, the Li||NCM811 cells with LiHMDS show a cycling life of 500 times at 60 °C with average coulombic efficiency of 99.11% and capacity retention of 66.02% (Fig. 1d). The cycling performances of Li||NCM811 cells with LiHMDS are well positioned in the literature when compared to other research works that investigate non-aqueous electrolyte solution additives in lab-scale Li||NCM811 cells (Supplementary Table 1). In sharp contrast, the Li||NCM811 cells with BE display lower average coulombic efficiency of 93.48% and present micro short circuit after 200 cycles. Moreover, the Li||NCM811 cells with LiHMDS exhibit a smaller polarization than that of the Li||NCM811 cells (Supplementary Fig. 4). The average discharge voltage of the Li||NCM811 cells with BE drops from 3.84 to 3.66 V after 300 cycles at 60 °C, while that of the Li||NCM811 cells with LiHMDS retains quite stable value up to 3.79 V (Supplementary Fig. 5). Furthermore, the discharge capacity of the Li||NCM811 cells with LiHMDS reaches 180.7 and 163.8 mA h g^−1^ at the rate of 900 mA g^−1^ and 1800 mA g^−1^ under 60 °C, respectively, which is much higher than that of Li||NCM811 cells with BE (167.5 and 139.4 mA h g^−1^) (Supplementary Fig. 6). Meanwhile, the LiHMDS enables 88.47% capacity retention of the Li||NCM811 cells with high NCM811 mass-loading of 10 mg cm^−2^ and thin lithium foil anode of 50 μm at the rate of 60 mA g^−1^ after 100 cycles under 60 °C, whereas the Li||NCM811 cells with BE present only 18.67% capacity retention for the same cycling conditions (Fig. 1e). This is due to that the side reactions between NCM811 and LMA with BE are more likely to occur than that with LiHMDS at elevated temperature and high cut-off voltage (Supplementary Figs. 7, 8), especially at the high cathode loading, which leads to larger consumption of LMA with only 50 μm thickness^33^. When the LiHMDS-containing electrolyte is tested in pouch cell configuration, the capacity retention of pouch cell is 93.4% after 75 cycles at 36 mA g^−1^ under 60 °C (Supplementary Fig. 9), confirming that the LiHMDS can hinder the side reactions between NCM811 and LMA with BE. Such cycling and rate improvements of Li||NCM811 cell with LiHMDS at high voltage and high temperature demonstrate the compatibility of LiHMDS with both NCM811 cathode and LMA. A stable interphase at the NCM811 | liquid electrolyte interface may be constructed by LiHMDS, which suppresses the phase transition on the NCM811 particle and side reactions of NCM811 with electrolyte.

In order to simulate possible localized overheated battery, alternative testing at low (i.e., 25 °C) and high (i.e., 60 °C) temperatures was carried out (Fig. 1f). After 50 cycles at different temperatures, the Li||NCM811 cells with LiHMDS show higher capacity retention of 95.54% and 95.82% at 25 °C and 60 °C than the Li||NCM811 cells with BE (88.08% and 93.81%, respectively). The electrochemical impedance spectroscopy (EIS) measurements show that the charge transfer resistance (*R*_ct_) of Li||NCM811 cells with BE increases from 10.74 Ω to 65.23 Ω after 100 cycles, while that of the Li||NCM811 cells with LiHMDS only increases from 5.11 Ω to 5.37 Ω after 100 cycles, supporting the speculation of the ability of LiHMDS to hinder the side reactions of NCM811 with electrolyte (Supplementary Fig. 10, Supplementary Table 2). In addition, we also assembled the graphite || NCM811 coin cells with LiHMDS, which present the capacity retention of 62.08% at 180 mA g^−1^ after 200 cycles under 60 °C, which is higher than that of graphite || NCM811 cells with BE (50.62%) (Supplementary Fig. 11). Furthermore, the Li||LiNi_0.5_Mn_1.5_O_2_ (LNMO) coin cells with LiHMDS also present better cycling performance at 147 mA g^−1^ than the Li||LNMO cells with BE (Supplementary Fig. 12).

### Effect of LiHMDS on the CEI of NCM811 cathode

**Fig. 2:**
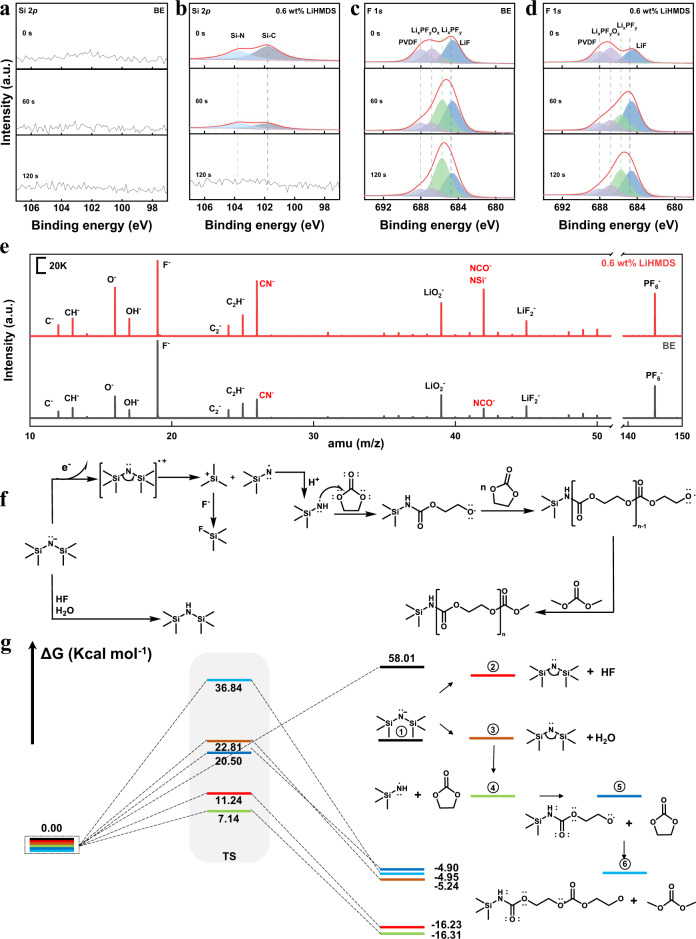
Surface component analysis of NCM811 cathode. Ex situ XPS measurements and analysis of NCM811 cathode retrieved from **a**, **c** Li||NCM811 cells and **b**, **d** Li||NCM811 cells with LiHMDS after 100 cycles at 60 °C, the charging/discharging current were 180 mA g^−1^/180 mA g^−1^. **a**, **b** Si 2*p*, **c**, **d** F 1*s*. **e** Ex situ TOF-SIMS analysis of NCM811 cathode retrieved from Li||NCM811 cells after 5 cycles at 60 °C, the charging/discharging current were 18 mA g^−1^/18 mA g^−1^. The cell was disassembled at fully discharged state. **f** Possible reaction mechanisms of LiHMDS. **g** Reaction energy diagram of LiHMDS.

The density functional theory (DFT) calculations present that the highest occupied molecular orbital (HOMO) level of the LiHMDS is −5.61 eV, which is higher than the other components of the electrolyte (Fig. 1g). The linear sweep voltammetry (LSV) curves also show that the LiHMDS is oxidized from 3.8 V to form CEI (Fig. 1h)^34^. These results indicate that the LiHMDS would be oxidized preferentially than electrolyte to form a new CEI on NCM811 cathode. The Si 2*p* spectra obtained from the ex situ X-ray photoelectron spectroscopy (XPS) measurements carried out on positive electrode sampled from cycled Li||NCM811 cells with LiHMDS show two peaks of Si-N (103.7 eV) and Si-C peak (101.8 eV) on the depth profiling compared to the XPS measurements carried out on positive electrode sampled from cycled from Li||NCM811 cells with BE (Fig. 2a, b), suggesting the deposition of oxidation products of LiHMDS on NCM811 surface. The signals of Si-N peak and Si-C peak only appear on the cathode surface suggest that the continuous decomposition of electrolyte solvent on the NCM811 cathode surface is inhibited once a thin and robust CEI layer is formed owning to the oxidation of LiHMDS. Compare to N 1*s* XPS spectra obtained from the ex situ measurements of positive electrode sampled from cycled Li||NCM811 cells with BE, the samples with LiHMDS show a N-Si peak (398.3 eV), which confirms that the LiHMDS participated in CEI formation (Supplementary Fig. 13a, b). In addition, the P 2*p* spectra of Li||NCM811 cell with LiHMDS show a presence of Li_x_PO_y_F_z_ (Supplementary Fig. 13c, d), which can scavenge the dissolved TMs (such as Co^2+^ and Ni^2+^) in the electrolyte to inhibit the TMs transfer to the LMA^35^. The lower intensity of Li_x_PF_y_ (686.8 eV) appeared in the F 1*s* spectra for the NCM811-based positive electrodes sampled from the cycled Li||NCM811 cells with LiHMDS indicates that the LiHMDS could suppress the decomposition of LiPF_6_^32,36,37^ (Fig. 2c, d). Furthermore, for Li 1*s* and F 1*s* XPS spectra, the LiF content in CEI using LiHMDS is higher than BE after Ar^+^ etching for 60 s and 120 s, which can enhance the strength of the CEI (Fig. 2c, d, Supplementary Fig. 13e, f). Therefore, the LiHMDS promotes the formation of more inorganic species such as LiF and Li_x_PO_y_F_z_ in the CEI (Supplementary Fig. 14). The O 1*s* spectra of the cycled NCM811 cathode sampled from Li||NCM811 cells with BE electrolyte show a peak at 530 eV^38^ (Supplementary Fig. 15a), which is ascribed to the metal oxide bonds (M-O) of NCM811, indicating that the uneven CEI formed on NCM811 particles using BE is unable to effectively passivate the highly active cathode surface (Supplementary Fig. 7b, c). In contrast, a weak M-O peak for the O 1*s* spectra and weak signals (Ni^2+^, Ni^3+^) for the Ni 2*p* spectra on the surface of NCM811 cathode sampled from Li||NCM811 cells with LiHMDS before etching appear (Supplementary Fig. 15b–d), indicating that a uniform CEI was formed on NCM811 surface. The O 1*s* spectra of the NCM811 cathode sampled from Li||NCM811 cell with LiHMDS on the depth profiling after etching for 120 s is similar to that of the pristine NCM811 particle, which indicates that LiHMDS is preferentially oxidated before electrolyte solvents (Supplementary Fig. 16).

Ex situ time-of-flight secondary ion mass spectrometry (TOF-SIMS) measurements were carried out on cycled positive electrodes to further identify the CEI components (Fig. 2e). The species with CN^-^ and NCO^-^ (NSi^-^) appear at *m/z* = 26 and *m/z* = 42, respectively, and their intensities for Li||NCM811 cell with LiHMDS are higher than those of Li||NCM811 cell with BE, confirming that the LiHMDS was oxidized on the surface of NCM811 cathode. We illustrate the possible two-stage reaction mechanisms of LiHMDS in Fig. 2f. First, the LiHMDS can be preferentially oxidized to form the radical anions, and then the radical anions capture the protons produced by the decomposition of carbonate solvents during cycling process and form the final radicals, which would polymerize with the EC and terminated by dimethyl carbonate (DMC) or ethyl methyl carbonate (EMC), thus forming a thin and uniform CEI film on the cathode surface^39^. Second, the LiHMDS can capture HF and trace amount of H_2_O in the electrolyte to form HMDS and other inorganic products (LiF and LiOH). We calculated the reaction energies (ΔG) of LiHMDS with HF and H_2_O by DFT method. The energies of LiHMDS reacted with HF and H_2_O are −16.23 and −5.24 kcal mol^−1^, respectively. These results suggest that the LiHMDS can react spontaneously with HF and H_2_O. In addition, the intermediates also can continue to spontaneously react with HF and H_2_O, forming radicals to initiate the ring-opening polymerization reaction of EC (Fig. 2g).

### Effect of LiHMDS on the structural evolution of NCM811

**Fig. 3:**
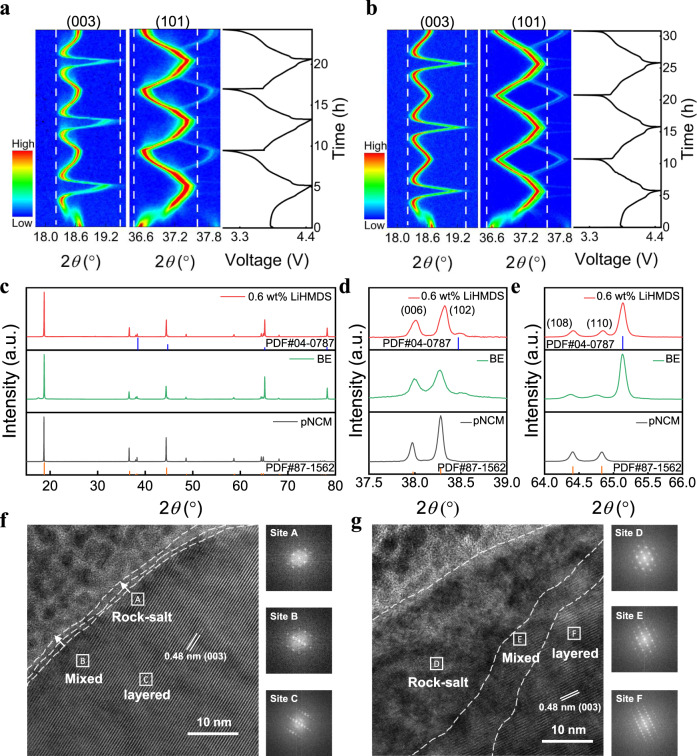
Structure characterizations of NCM811 cathode. Operando XRD characterization for NCM811 cathode during the initial three charge-discharge cycles using **a** BE and **b** BE with LiHMDS. the charging/discharging current were 36 mA g^−1^/36 mA g^−1^. **c**–**e** Ex situ XRD measurements of NCM811 cathode before and after 100 cycles in BE and BE with LiHMDS at 60 °C, the charging/discharging current were 180 mA g^−1^/180 mA g^−1^. Ex situ TEM and FFT images of cycled NCM811 cathodes using **f** BE with LiHMDS and **g** BE after 100 cycles at 60 °C, the charging/discharging current were 180 mA g^−1^/180 mA g^−1^. The cell was disassembled at fully discharged state.

Operando X-ray diffraction (XRD) measurements were carried out to investigate the structural evolution of NCM811 cathode with different electrolytes during charge-discharge process (Fig. 3a, b). The (003) and (101) peaks intensity and the Brag angle of NCM811 cathode cycled in BE change markedly during the phase transition from H1 phase to M phase at ~3.8 V (Supplementary Fig. 17). After transition to the H2 phase, the (003) peak starts to shift left and then right, which corresponds to a phase transition from H2 to H3 phase with a reduced interlayer spacing^40^. Left/right (003) peak shifts related to lattice expansion/contraction. Noticeably, the change of the (003) peak position of cycled NCM811 cathode using LiHMDS is much less than that using BE, which means that the change of lattice parameter *c* is hindered by LiHMDS during the charge process^40^. In addition, the (101) peak using LiHMDS related to the lattice parameter *a* shifts less during the whole charge process. These results confirm that the cycled NCM811 cathode with LiHMDS presents a more reversible structural evolution, which maintains well during cycle. Moreover, the XRD patterns show that the separation of (006)/(102) and (108)/(110) peaks is more clear for the cycled NCM811 with LiHMDS (Fig. 3c–e), indicating that the structure transformation from layered to rock salt phase is successfully suppressed^41^. The lower intensity ratio of R-factor (I(006)+I(012))/I(101) also suggests the good structure stability of cycled NCM811 with LiHMDS (Supplementary Fig. 18)^42^.

The improved structural stability of NCM811 during cycling with LiHMDS is attributed to the optimized CEI formed by LiHMDS. Ex situ TEM measurements of cycled positive electrodes show that a uniform and amorphous CEI with thickness of 5 nm was formed on the surface of NCM811 using LiHMDS after 100 cycles at 60 °C (Supplementary Figure 7a), much more uniform than that formed using BE (3 nm ~ 20 nm) (Supplementary Fig. 7b, c). The formation of the nanometric and uniform CEI indicates that the unwanted species like HF in the electrolyte have been sequestrated completely by the LiHMDS, and thus no damage on the CEI occurs. Consequently, the NCM811 particles are protected by the CEI from possible side reaction with the electrolyte, which can avoid the reduction of the high valence Ni^4+^ in the delithiated NCM811 and suppress the phase transition from layered structure to rock-salt phase (Fig. 3f). Ex situ TEM measurements of cycled positive electrodes show that the layered structure is well preserved from the surface to the bulk after cycling with LiHMDS and negligible rock-salt phase formation was formed. On the contrary, a thicker rock-salt phase is observed on the surface region of the NCM811 particle cycled in BE (Fig. 3g)^40^.

We also conducted an accelerated degradation test by holding the fully charged Li||NCM811 cells at 4.5 V (Supplementary Fig. 19). The Li||NCM811 cells with LiHMDS present a quasi-steady-state leakage current of ~5 μA after 2 h hold, while the leakage current of cell using BE slowly decreases and reaches a minimum value of 15 μA after 12 h hold. The larger 4.5 V float-test leakage current suggests that more side reactions occur for the cell cycled with BE than that with LiHMDS. This result further proves that a diminished side reactions rate and passivated NCM811 cathode surface using LiHMDS are achieved due to formation of a thin and robust CEI^12^. Furthermore, the ex situ scanning electron microscopy (SEM) micrographs of cycled positive electrodes show intergranular macro cracking on the NCM811 particle with BE, while the cycled NCM811 cathode with LiHMDS did not experience intergranular cracking and maintain the structure (Supplementary Figs. 20 and 21). The intergranular macro cracking were generated owning to the internal strain inside the particles induced by volume change during the charge-discharge process^43^. As a result, more fresh interfacial areas are exposed to electrolyte, resulting in rapid capacity fade and formation of thick CEI on NCM811 particle with BE.

### Effect of LiHMDS on the solid electrolyte interphase (SEI) of LMA

The LiHMDS is also beneficial for the formation a stable SEI and improve the Li stripping/plating process on the LMA. Via ex situ measurements, the P 2*p* and Ni 2*p* XPS spectra of LMA cycled with LiHMDS and BE show different components. A relatively high fraction of Li_x_PF_y_, Li_x_PO_y_F_z,_ NiF_2_ and Ni metal were observed on the cycled LMA surface with BE (Supplementary Fig. 22a–c). The formation of Li_x_PF_y_ and Li_x_PO_y_F_z_ were attributed to the decomposition of LiPF_6_ salt, and NiF_2_ was formed due to the HF induced dissolution of Ni in NCM811. The Ni metal in the SEI layer results from the reduction of dissolved Ni^2+^ by lithium metal. The TM elements on the LMA surface leads to the SEI damaging^44^, consumption of electrically active Li and increase of cell impedance^12^. In sharp contrast, a smaller amount of Li_x_PF_y_, Li_x_PO_y_F_z,_ NiF_2_ and Ni element were found on the cycled LMA with LiHMDS (Supplementary Fig. 22d, e), indicating that the LiHMDS inhibits the lithium salt decomposition and suppresses the dissolution of TM ions^17^. In addition, there is only LiF examined in the cell with LiHMDS (Supplementary Fig. 22f) compared to the LMA cycled in the BE (Supplementary Fig. 22b). This indicates that the SEI derived from LiHMDS consists of more LiF-like inorganic components, which benefits for the cycling stability of Li metal batteries^12^. Moreover, the surface of cycled Li metal electrode using LiHMDS after 100 cycles at 180 mA g^−1^ and 60 °C presents a more compact structure than that using BE, indicating the stability of the SEI using LiHMDS. The cross-sectional SEM images show that the thickness of loose structure on cycled Li metal electrode (~95 μm) is thicker than that using LiHMDS (32 μm) due to the side reactions between Li and BE^12^ (Supplementary Fig. 8c, d). This behavior also can be found on the thin Li electrode of 50 μm after 10 cycles at 180 mA h g^−1^ (Supplementary Fig. 23).

**Fig. 4:**
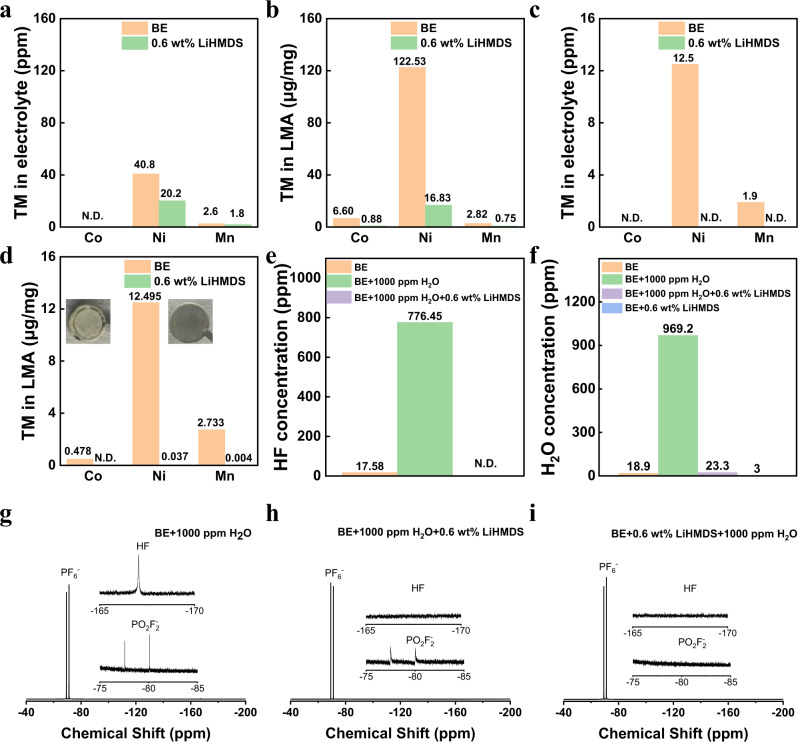
Ex situ measurements of the cycled electrolyte solutions and Li metal electrodes. ICP-MS analysis of cycled **a** electrolyte and **b** LMA after 100 cycles at 60 °C, the charging/discharging current were 180 mA g^−1^/180 mA g^−1^, the cell was disassembled at fully discharged state. ICP-MS analysis of **c** electrolyte and **d** LMA after rest at open circuit voltage for 7 days at 60 °C. **e** HF concentration of BE, BE + 1000 ppm H_2_O and BE + 0.6 wt% LiHMDS + 1000 ppm H_2_O**. f** H_2_O concentration of BE, BE + 0.6 wt% LiHMDS, BE + 1000 ppm H_2_O and BE + 0.6 wt% LiHMDS + 1000 ppm H_2_O. ^19^F NMR spectra of the solution of **g** BE + 1000 ppm H_2_O, **h** BE + 1000 ppm + 0.6 wt% LiHMDS and **i** BE + 0.6 wt% LiHMDS + 1000 ppm H_2_O.

The content of TM elements in the SEI layer formed in electrolyte with and without LiHMDS is further verified by inductively coupled plasma optical emission spectrometer (ICP-OES) measurement. As shown in Fig. 4a, b, the content of Ni, Co, and Mn in electrolyte and LMA from Li||NCM811 cell with LiHMDS after 100 cycles at 180 mA g^−1^ under 60 °C is much lower than that of Li||NCM811 cell. In particular, the Ni content on the surface of LMA from the Li||NCM811 cell with LiHMDS is 16.83 µg mg^−1^, much lower than that from Li||NCM811 cell (122.53 µg mg^−1^). The suppressed TM ions dissolution can be attributed to the formation of a robust CEI on the NCM811 by LiHMDS. In order to further understand the CEI formation process, ex situ nuclear magnetic resonance (NMR) measurements of the cycled electrolyte was used to verify the LiHMDS content after 3 cycles at 36 mA g^−1^ under 60 °C. As shown in Supplementary Fig. 24, the HMDS^-^ derivatives signals could be clearly observed before cycling, but it is found that the peaks disappear after 3 cycles 36 mA g^−1^ under 60 °C. In addition, we did not find any evidence of Mn(HMDS)_2_ or Ni(HMDS)_2_. Above results prove that the LiHMDS has been completely consumed after 3 cycles. Since the electrolyte decomposition would be accelerated at elevated temperature, we also measured the TM ions concentration in electrolyte and on the LMA surface after the cells were stored in oven at 60 °C for 7 days. It is found that no TM ions were observed in the electrolyte with LiHMDS and very few TM ions were detected on the LMA surface (Fig. 4c, d), which are much lower than that of the Li||NCM811 cells. Accurate determination of the H_2_O and HF content in the electrolyte is essential for understanding the role of LiHMDS. As shown in Fig. 4e, the concentration of HF increases dramatically from 17.58 ppm to 776.45 ppm after adding 1000 ppm H_2_O to the BE as measured by acid-base titration method. But after LiHMDS was added to the solution consisting of BE and 1000 ppm H_2_O, the solution turns to alkaline, which means that the HF was sequestrated by the LiHMDS. Karl Fischer titration method was further adopted to accurately determine the H_2_O amount. As shown in Fig. 4f, the H_2_O content of BE is 18.9 ppm, while after LiHMDS was dissolved in the BE and rest for 12 h, the H_2_O content sharply decreases to 3 ppm. Even when the H_2_O content raises to 969.2 ppm by dropped 5 μL H_2_O into 5 mL BE, most of H_2_O can be eliminated by LiHMDS. The residual trace H_2_O may be caused by moisture in the environment during examination of the H_2_O content. To further understand the interaction structures of HF and H_2_O with electrolyte, the binding energy and bond length of H_2_O-PF_6_^−^, HF- HMDS^−^, and H_2_O-HMDS^-^ were studied by DFT calculation (Supplementary Fig. 25a, b). It is seen that the HF presents a high binding energy with LiHMDS (Supplementary Fig. 25c), which is almost 2.13 and 10.57 times higher than that of H_2_O-HMDS^-^ and H_2_O-PF_6_^−^. These results support the claim about the LiHMDS capability to eliminate the HF and H_2_O in the electrolyte, which is significant and crucial to achieve higher capacity retentions of the Li||NCM811 cells with LiHMDS under harsh conditions.

## Discussion

### Mechanisms of LiHMDS for removing HF

To understand the reaction mechanism of LiHMDS with HF and H_2_O, the ^19^F NMR measurements on various electrolyte solutions were carried out. After adding 1000 ppm H_2_O into the BE and stored at 25 °C for 1 day, the ^19^F NMR spectra shows two doublet peaks and one singlet peak (Fig. 4g). The doublet peak located at −71.2 ppm and −79.3 ppm is associated with the PF_6_^−^ in LiPF_6_. The other doublet peak (−77.5 and −80.1 ppm) and the singlet peak (−167.1 ppm) are corresponding to the PO_2_F_2_^-^ and HF, respectively^45^. Moreover, we added 1000 ppm H_2_O into BE with LiHMDS and carried out the ^19^F NMR measurements. Intriguingly, after adding LiHMDS into BE with 1000 ppm H_2_O, the peak of HF was not detectable (Fig. 4h) and only one doublet peak of PF_6_^-^ can be observed (Fig. 4i). Above results demonstrate that the LiHMDS reacts with H_2_O more actively than the LiPF_6_ and can eliminate HF in the electrolyte. The thermal stability of BE and electrolyte with LiHMDS in Li||NCM811 cells at high temperature was also evaluated by ^19^F NMR measurements. The Li||NCM811 cells were assembled and stored at 60 °C for 7 days without cycling and disassembled in an Ar-filled glovebox before testing. As shown in Supplementary Fig. 26a, the peak of HF was not detected in BE, which might be ascribed to the reactions of HF with LMA and NCM811 particles. However, the byproduct of PO_2_F_2_^-^ derived from the thermal decomposition of LiPF_6_ could be clearly observed, while which cannot be found in the electrolyte with LiHMDS (Supplementary Fig. 26b), further demonstrating that the LiHMDS could significantly improve the thermal stability of electrolyte. We also examined the cycling performance of the Li||NCM811 cells with 1000 ppm H_2_O in the electrolytes at 180 mA g^−1^ under 60 °C (Supplementary Fig. 27a). The coulombic efficiency of the full cells cycled using BE with 1000 ppm H_2_O is only 37.41% at the first cycle and the charge curve is abnormal (Supplementary Fig. 27b), suggesting the severe side reactions between LiPF_6_ and H_2_O during charging. In sharp contrast, the Li||NCM811 cells with LiHMDS and 1000 ppm H_2_O presents a higher initial coulombic efficiency (68.37%) and delivers a discharge capacity of 217.9 mA h g^−1^.

**Fig. 5:**
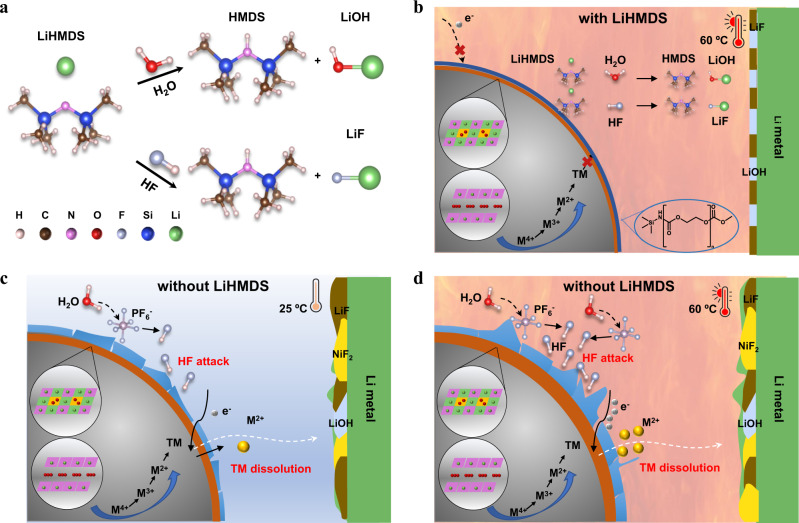
Function schematic illustration of LiHMDS in Li||NCM811 cells. **a** Reaction mechanism of LiHMDS with HF and H_2_O. **b**–**d** Working mechanism of Li||NCM811 cells with/without LiHMDS cells at 25 °C and 60 °C.

From the experimental and theoretical results, the function of LiHMDS is illustrated schematically in Fig. 5. Owning to the high binding energy of HMDS^-^ with H_2_O- and HF and the intrinsic organic base nature of LiHMDS, the LiHMDS could capture H_2_O and HF quickly (Fig. 5a). Therefore, in the Li||NCM811 cells with LiHMDS at 60 °C, the LiHMDS not only captures HF and H_2_O in the electrolyte, but also constructs uniform, thin and robust CEI layer before oxidation of electrolyte, which inhibits the TM ions dissolution and improves the cyclic performance at high voltage and temperature (Fig. 5b). However, in the Li||NCM811 cells at 25 °C, trace amount of water reacts with the LiPF_6_ to generate HF, which damages the CEI derived from oxidation of electrolyte that leads to the TM ions dissolution and causes the capacity fade of NCM811 and cation ion mixing (Fig. 5c). At 60 °C, the side reactions such as LiPF_6_ decomposition, TM dissolution and their shuttle effect in the Li||NCM811 cells are more serious than those in 25 °C, which leads to worse cell performance (Fig. 5d).

In summary, we investigated a non-aqueous electrolyte additive of LiHMDS with low oxidation potential for long cycling Li||NCM811 batteries at both high voltage of 4.5 V and high temperature of 60 °C. The Li||NCM811 batteries with 0.6% LiHMDS cycled between 3.0 and 4.5 V demonstrates a capacity retention of 73.92% after 1000 cycles at 25 °C and 66.02% after 500 cycles at 60 °C and180 mA g^−1^. The LiHMDS not only efficiently depletes HF and H_2_O (even 1000 ppm) in the electrolyte, but also is preferentially oxidized prior to electrolyte to construct a thin, uniform and robust CEI on NCM811 cathode surface, which can suppress the side reactions with electrolyte, phase transformation of NCM811 from layered structure to rock-salt phase and endows NCM811 cathode with good thermal shock resistance. As a result, the TM ions dissolution of NCM811 and their attack for SEI on the LMA are successfully suppressed, which improves the cycling stability of Li||NCM811 batteries at both high voltage and high temperature.

## Methods

### Materials

The commercial LiNi_0.8_Co_0.1_Mn_0.1_O_2_ (NCM811, D_50_ = 3.77 μm), LiNi_0.5_Mn_1.5_O_2_ (LNMO, D_50_ = 11.61 μm), polyvinylidene difluoride (PVDF5130, Mw = 1,200,000 Da, particle size: 100 μm, 99.5%), Super P (particle size: 40–50 nm, 99.5%), lithium polyacrylate (PAALi, 4 wt% in water), Graphite (D_50_ = 19.2 μm, 99.5%), Al foil (thickness: 10 μm, 99.9%), Cu foil (thickness: 10 μm, 99.9%), separator (Celgard 2500, thickness: 25 μm, monolayer PP membrane, pore size: average diameter 0.064 μm, porosity: 55 ± 5%), Coin cell components (CR2032, spacer: 15.8 × 1 mm, spring: 15.4 × 1.1 mm) and the baseline electrolyte (BE) (1 M LiPF_6_ in EC, EMC and DMC (1:1:1 by volume), (H_2_O < 20 ppm)) were purchased from Guangdong Canrd New Energy Technology Co., Ltd. Lithium hexamethyldisilazide (LiHMDS, 97%) came from Sigma-Aldrich. N-methyl-2-pyrrolidone (NMP, AR) and DMC (99%, (H_2_O < 20 ppm)) were purchased from Shanghai Aladdin Biochemical Technology Co., Ltd. Lithium foil (thickness: 450 μm, diameter: 15.6 μm) and double side coated Cu foil (20 μm Li each side and the thickness of Cu is 10 μm, the whole thickness of Li anode is 50 μm) came from China Energy Lithium Co., Ltd. Dimethyl sulfoxide-d6 (DMSO-d6, 0.6 mL, 99.9%, +0.03% V/V tetramethyl silane (TMS)) came from Shanghai Macklin Biochemical Technology Co., Ltd. All materials were used as received. To prepare electrolytes with 0.6 wt% LiHMDS, 16.7 mg LiHMDS was dissolved in 2 mL BE by magnetic stirring at 25 ± 2 °C for 30 min.

### Fabrication of cells

NCM811 and LNMO cathode was prepared by following steps. The mixture of 0.8 g (80 wt%) single crystal NCM811 (or LNMO) powder, 0.1 g (10 wt%) PVDF5130 and 0.1 g (10 wt%) Super P was manually grinded in an agate mortar for 10 min in an air environment and then dispersed in 2 mL NMP by magnetic stirring for 4 h to form a slurry. Then the slurry was casted on an Al foil and dried at 120 °C for 2 h under vacuum. Graphite anode was prepared by following steps. 1 g (4 wt%) PAALi was diluted by 1 mL H_2_O, then 0.15 g (1.5 wt%) Super P was added to the solution and stirring for 30 mins, and added 0.945 g (94.5 wt%) graphite in the emulsion and stirring for 4 h to form a slurry. Then the slurry was casted on a Cu foil and dried at 80 °C for 8 h under vacuum. The CR2032 Li||NCM811, Graphite||NCM811 and Li||LNMO coin cells were assembled in an Ar-filled glove box (O_2_ and H_2_O < 0.1 ppm) using 50 μL BE with/without LiHMDS. The thickness of lithium foil is 450 μm and the diameter is 15.6 mm. The diameter of cathode is 12 mm, and the thickness of separator is 25 μm. In the coin cell, 2 spacers and a spring was used in the cell. The separator was Celgard2500. For Li||NCM811 pouch cell, two single side coated cathode (mass loading: ~10 mg cm^−2^, 4 cm × 3.5 cm) and one double coated anode (4.3 cm × 4 cm) were stacked one by one and separated by Celgard 2500 (4.5 cm × 4.5 cm), the electrolyte volume is 200 μL. We assembled in a dry room whose dew point is −50 °C.

### Electrochemical measurements

The LSV measurements of Li||stainless steel coin cells with different electrolytes were examined from 2.5 to 5.5 V vs. Li at a scanning rate of 0.5 mV s^−1^ under 60 °C using a VMP3 multichannel electrochemical station (Bio Logic Sciensce Instruments, France). The cyclic voltammetry (CV) measurements of Li||NCM811cells were examined between 3 and 4.5 V at a scanning rate of 0.05 mV s^−1^ under 25 °C and 60 °C using a VMP3. The electrochemical impedance spectroscopy (EIS) of Li||NCM811cells was performed from 7 MHz to 10 mHz at an amplitude of 5 mV, 6 data points per decade, and the 3.95 V of charging progress (after charge to 3.95 V, a constant-voltage charging was applied until the specific current is lower than 9 mA g^−1^, and rest for 2 h) applied before carrying out the EIS measurements at 60 °C using a VMP3. Cycling and rate performances of Li||NCM811cells were measured at 25 °C and 60 °C on a battery test system (LAND CT-2001A) with a voltage range from 3 to 4.5 V. The electrochemical floating test was performed in Li||NCM811cells with different electrolyte. The cells were first charged to 4.5 V at 18 mA g^−1^ and then maintained for 12 h with the current monitored by LAND battery test system. For all electrochemical measurement, four cells were measured at the same condition. And the specific current and specific capacity refers to the mass of the active material in the cathode. The electrochemical energy storage tests at various temperatures were carried out in a constant temperature chamber.

### Materials characterization

The X-ray diffraction (XRD) measurements of the samples were carried out on a Rigaku Smartlab with Cu-Kα radiation. The operando XRD test during charging and discharging rate of 36 mA g^−1^were performed at 60 °C and diffraction patterns were collected every 8 min. The morphologies and structures of NCM cathode and Li anode were characterized by a scanning electron microscope (SEM, HITACH S4800) with energy dispersive spectroscopy (EDS) and a field emission transmission electron microscope (FE-TEM, FEI Tecnai F30). X-ray photoelectron spectroscopy (XPS) measurements was collected on a PHI 5000 VersaProbe II instrument. The ^19^F and ^1^H nuclear magnetic resonance (NMR) data were collected with NMR spectrometer (ADVANCED III 400 MHz, Bruker, Switzerland) with DMSO-*d6* solvent. The type of NMR-Tube is WILMAD WF-1000-7 5 mm. To prepare the samples for NMR, we assembled the Li||NCM811 coin cells with/without LiHMDS, which were stored at 60 °C for 7 days. Then we disassembled the cells and washed the separator with 0.6 mL DMSO-d6 and collected the solution for NMR measurement in the Ar-filled glovebox. The transition metal (TM) concentration of electrolyte and TM content of Li anode was measured by inductively coupled plasma optical emission spectrometer (ICP-OES, SpectroArcosII MV, Germany). The cycled LMA was dissolved in 10 mL 1 M HCl, and the TM content on the surface of LMA is calculated by the mass of TM elements in the solution over the mass of active material in the NCM811 cathode. For the TM concentration of electrolyte, we soaked the cathode, separator and anode in 5 mL DMC for 5 min and collected all the liquid in the Ar-filled glovebox, then the solvent was evaporated in oven under 80 °C, and the residues was dissolved in 1 mL 1 M HCl and then dilute to 10 mL solution with 9 mL deionized H_2_O using 10 mL volumetric flask. For the TM content of LMA surface, the washed LMA was dissolved in 2 mL 1 M HCl and then dilute to 10 mL solution with deionized H_2_O using 10 mL volumetric flask. The CEI component data was collected by time-of-flight secondary ion mass spectrometry (ToF-SIMS, PHI nanoTOF II, 30 keV, 2 nA, and the raster size is 60 × 60 μm.). The cross-sectional NCM cathode is investigated by ion milling system (IMS, HITACH IM4000 plus). For HF content test, BE + 1000 ppm H_2_O + 0.6 wt% LiHMDS means that we added 1000 ppm to BE and the obtained solution was stored for 4 h, and then 0.6 wt% LiHMDS was added afterward and stored for 4 h again. The purpose of this experiment is to confirm the role of LiHMDS for eliminating HF. BE + 0.6 wt% LiHMDS + 1000 ppm H_2_O means that after 0.6 wt% LiHMDS was dissolved in BE, 1000 ppm H_2_O was added to the solution and the LiHMDS can absorb the H_2_O. The HF and H_2_O content of electrolyte was measured by acid-base titration method and Karl Fischer titration method, and sample preparation of electrolyte is the same as ^19^F NMR measurement. Prior to analysis, the cells were disassembled in Ar-filled glove box (Mikrouna) (H_2_O < 0.1 ppm, O_2_ < 0.1 ppm) and the electrode surfaces were rinsed with 5 mL of DMC. After drying at 25 °C in glovebox for 10 min, the electrodes were transferred by the transfer sample holder with an Ar-filled to isolate the air.

### Computational methods

The highest occupied molecular orbital and lowest unoccupied molecular orbital (HOMO and LUMO) energy of EC, EMC, DMC, LiPF_6_ and LiHMDS was calculated by quantum-chemical calculations (based on density functional theory, B3LYP/6-311G (d, p)). The optimum structures were optimized by B3LYP method in combination with the 6-31G (d, p) basis set and the more precise energy was calculated by same method in combination with the 6-311 G++ (d, p) basis set. The vibration frequency and intrinsic reaction coordinate (IRC) analysis were adopted to confirm each transition state (TS) that connects both product and reactant in the same pathway at the same level. The stable structure between H_2_O and Li^+^, PF_6_^-^ and HDMS^-^ were obtained from the Molecular dynamics (MD) simulation with the force-fields of water model involving four charge sites (TIP4P)^46^ and the optimized potentials for liquid simulations (OPLS-AA)^47^ and assigned with RESP charges^48^.

### Reporting summary

Further information on research design is available in the Nature Portfolio Reporting Summary linked to this article.

## Supplementary information

Supplementary Information

Reporting Summary

## Data Availability

The authors declare that all the relevant data are available within the paper and its Supplementary Information file or from the corresponding author upon reasonable request.
