# Supplementary material for: Lithium hexamethyldisilazide as electrolyte additive for efficient cycling of high-voltage non-aqueous lithium metal batteries
Source: Nat Commun. 2022 Nov 15;13:6966. doi: 10.1038/s41467-022-34717-4 (PMC9666536; doi:10.1038/s41467-022-34717-4)
Supplement: Supplementary file 1 — Supplementary Information [file 41467_2022_34717_MOESM1_ESM.pdf]

Supplementary Information

Lithium hexamethyldisilazide as electrolyte additive for efficient cycling of high-voltage non-aqueous lithium metal batteries

Danfeng Zhang[1,2,6], Ming Liu[1,6], Jiabin Ma[1,2], Ke Yang[1,2], Zhen Chen[1], Kaikai Li[3], Chen Zhang[1], Yinping Wei[1,2], Min Zhou[4], Peng Wang[1], Yuanbiao He[1], Wei Lv[1], Quan-hong Yang[5], Feiyu Kang[1,2], Yan-Bing He[1]*

[1] Shenzhen All-Solid-State Lithium Battery Electrolyte Engineering Research Center, Institute of Materials Research (IMR), Tsinghua Shenzhen International Graduate School, Tsinghua University, Shenzhen 518055, China.

[2] School of Materials Science and Engineering, Tsinghua University, Beijing, 100084, China.

[3] School of Materials Science and Engineering, Harbin Institute of Technology, Shenzhen, 518055, China.

[4] School of Pharmacy, East China University of Science and Technology, Shanghai, 200237, China

[5] Nanoyang Group, State Key Laboratory of Chemical Engineering, School of Chemical Engineering and Technology, Tianjin University, Tianjin, 300072 China

[6] These authors contributed equally: Danfeng Zhang, Ming Liu.

Correspondence and requests for materials should be addressed to Y.-B. H. (email: he.yanbing@sz.tsinghua.edu.cn)

Supplementary Figures

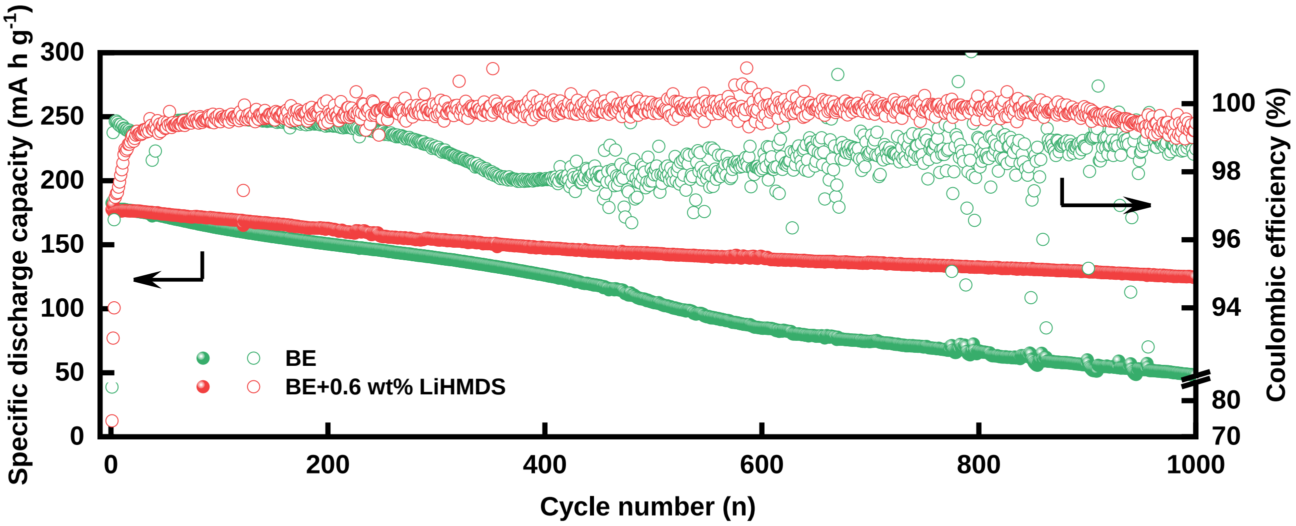

Supplementary Figure 1 Cycling performance of Li||NCM811 cells with and without LiHMDS additives at 90 mA g^{-1} at 25 °C between 2.8 V and 4.3 V.

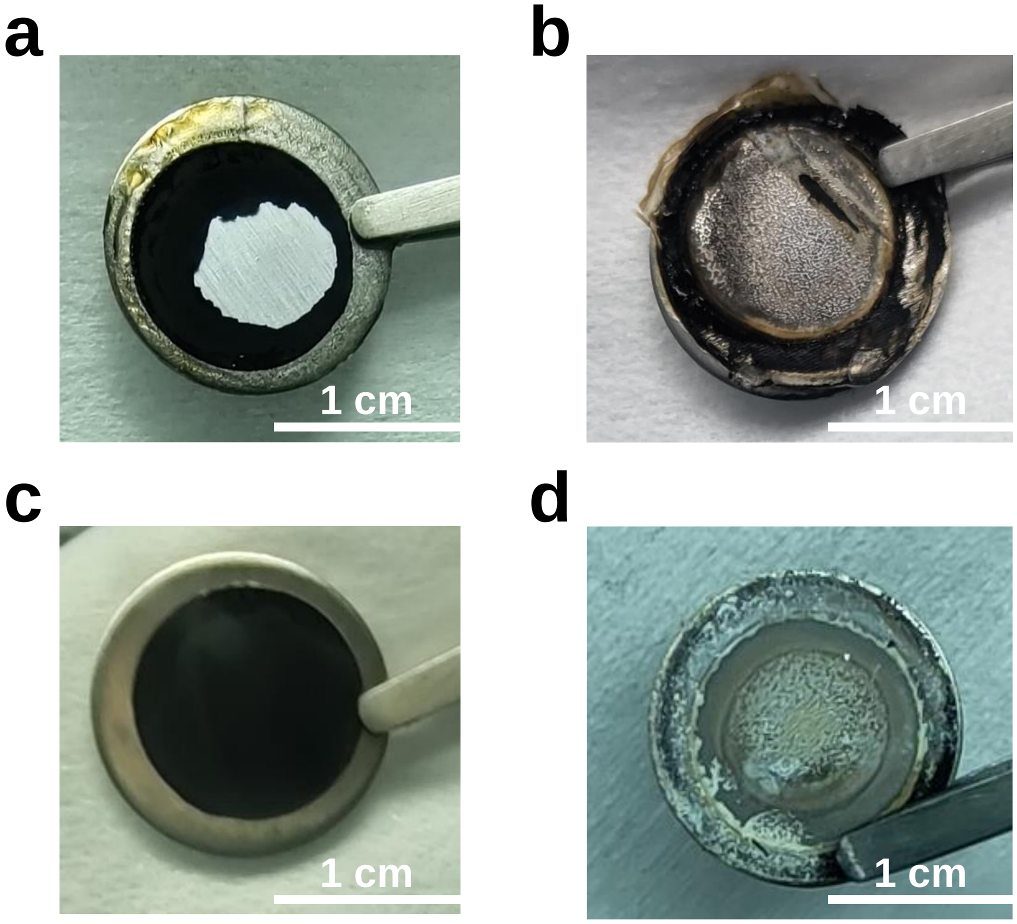

Supplementary Figure 2 Optical photo of components of Li||NCM811 cells using (**a, b)** BE and (**c, d)** 0.6 wt% LiHMDS after 400 cycles at 180 mA g^{-1} under 25 °C between 2.8 and 4.3 V. (**a, c)** Cathode, (**b, d)** Lithium metal anode. The cell was disassembled at fully discharged state.

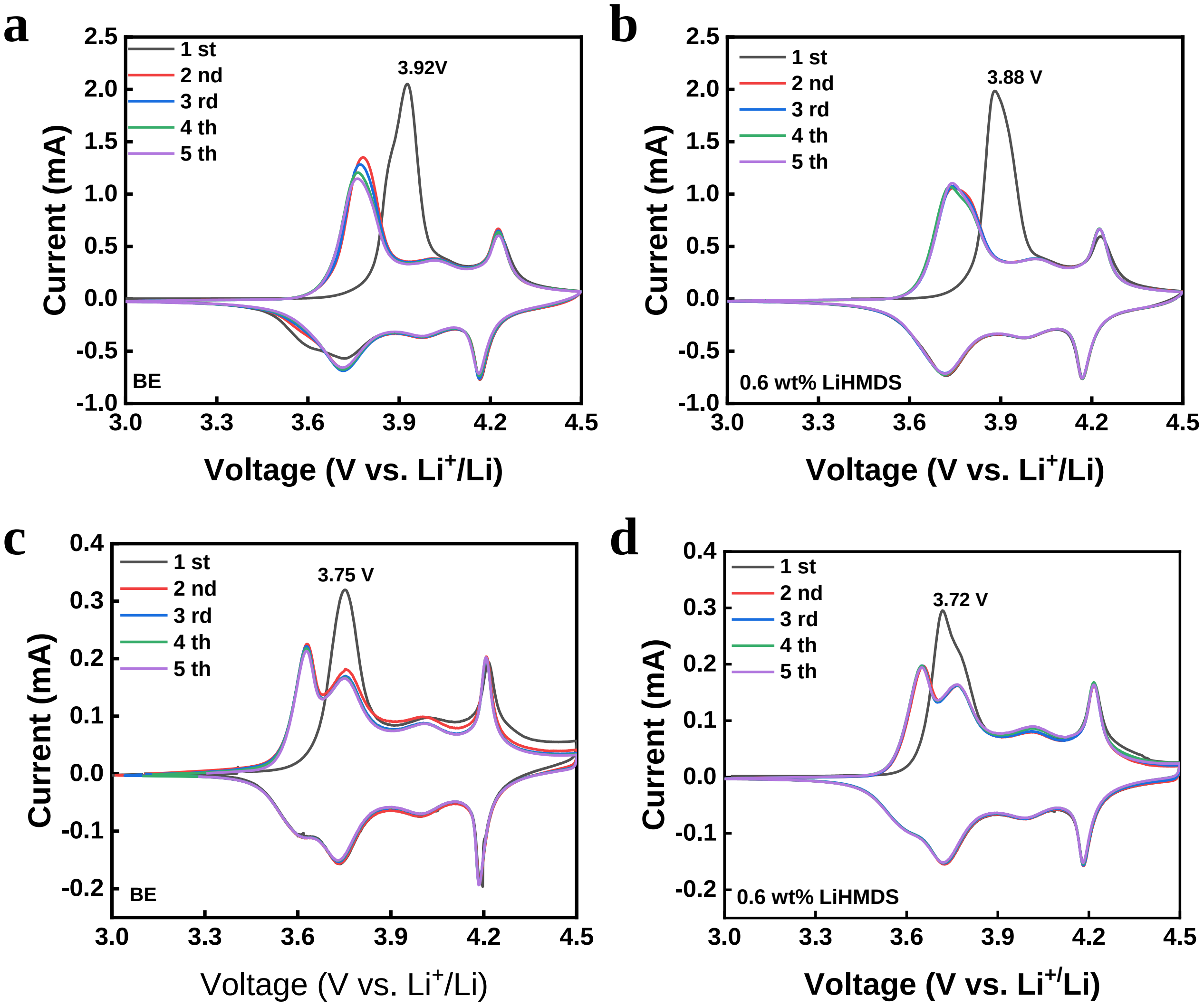

Supplementary Figure 3 Cyclic voltammetry curves of Li||NCM811 cells and Li||NCM811 cells with LiHMDS at (**a, b)** 25 °C and (**c, d)** 60 °C between 3 and 4.5 V. Sweep rate: 0.05 mV s^{-1}.

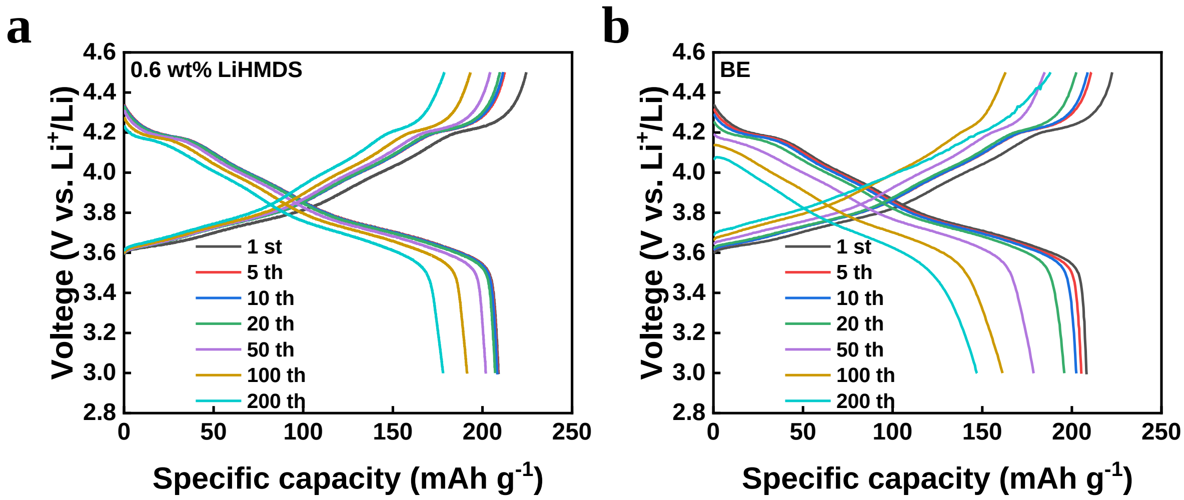

Supplementary Figure 4 Charge/discharge curves of Li||NCM811 cells at 60 °C with **(a)** LiHMDS and (**b)** BE at 180 mA g^{-1} under 60 ºC.

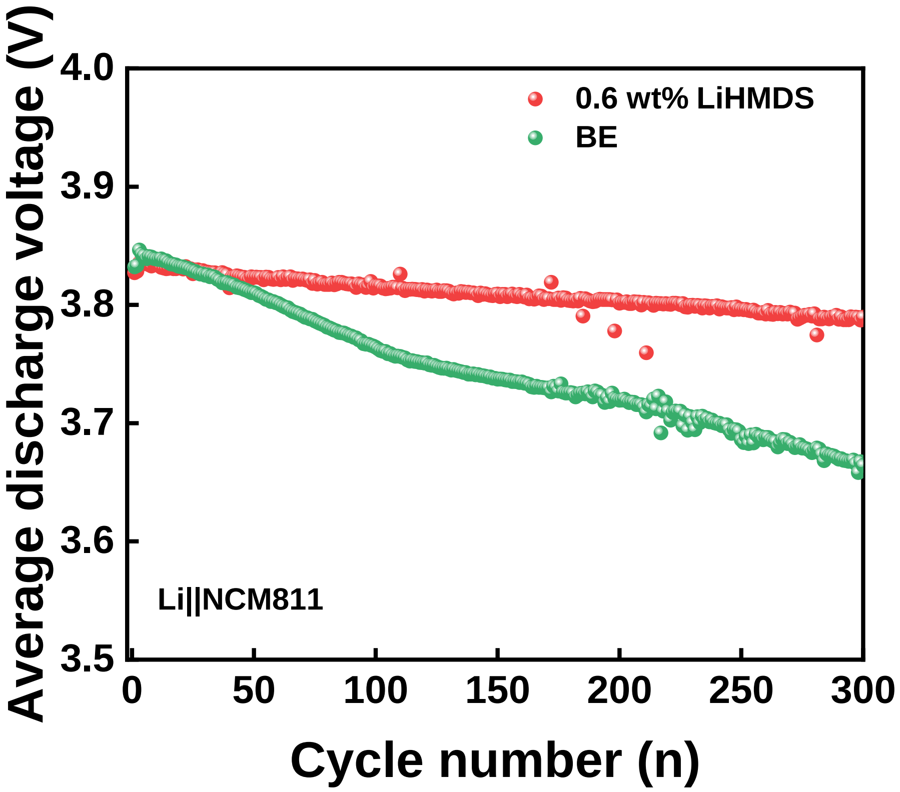

Supplementary Figure 5 Average discharge voltage of Li||NCM811 cells with and without LiHMDS additives electrolytes charged and discharged between 3.0 and 4.5 V at 180 mA g^{-1} under 60 °C.

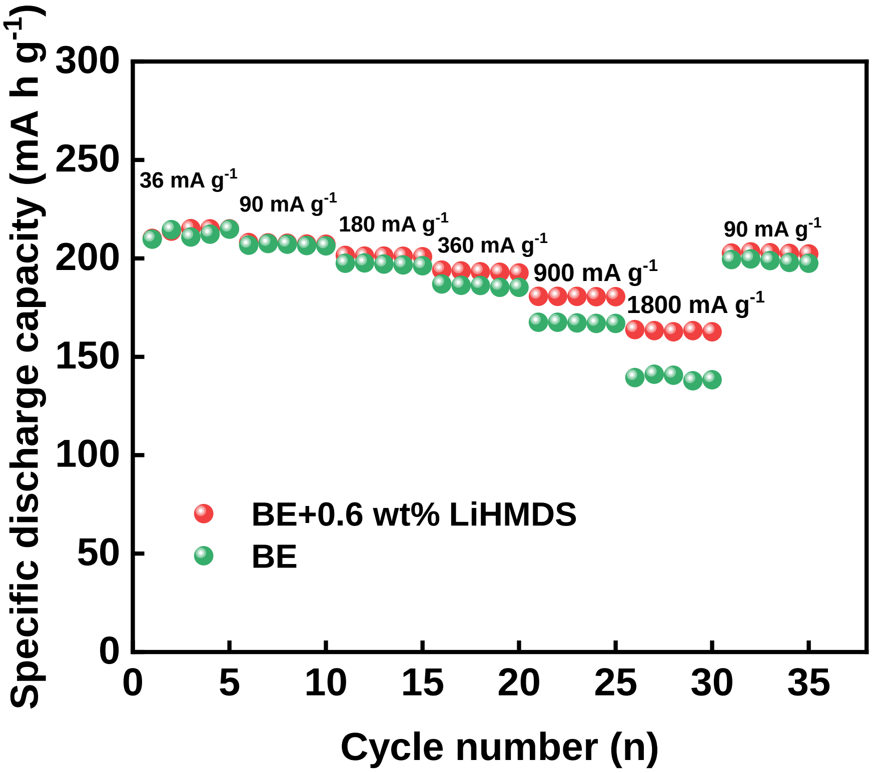

Supplementary Figure 6 Rating performance of Li||NCM811 cells at 25 °C.

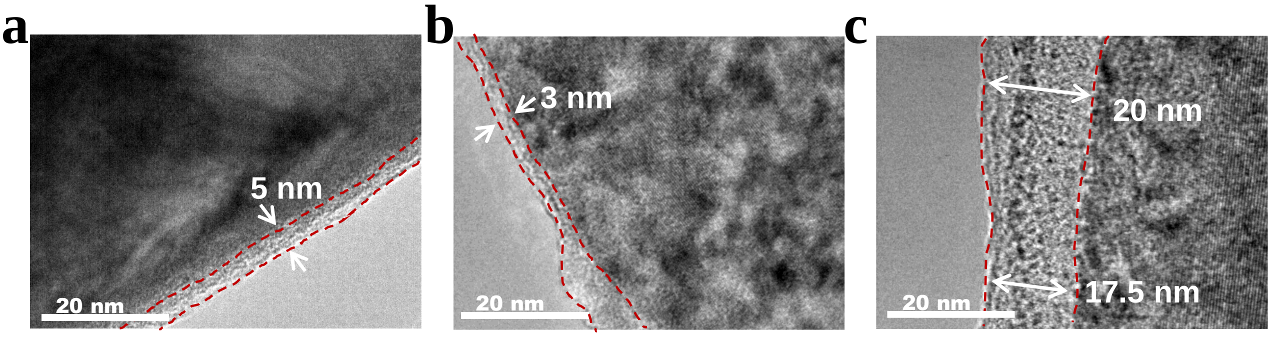

Supplementary Figure 7 Ex situ TEM images of NCM cathode retrieved from **(a)** Li||NCM811 cells with LiHMDS and **(b, c)** Li||NCM811 cells with LiHMDS after 100 cycles at 180 mA g^{-1} under 60 °C. The cell was disassembled at fully discharged state.

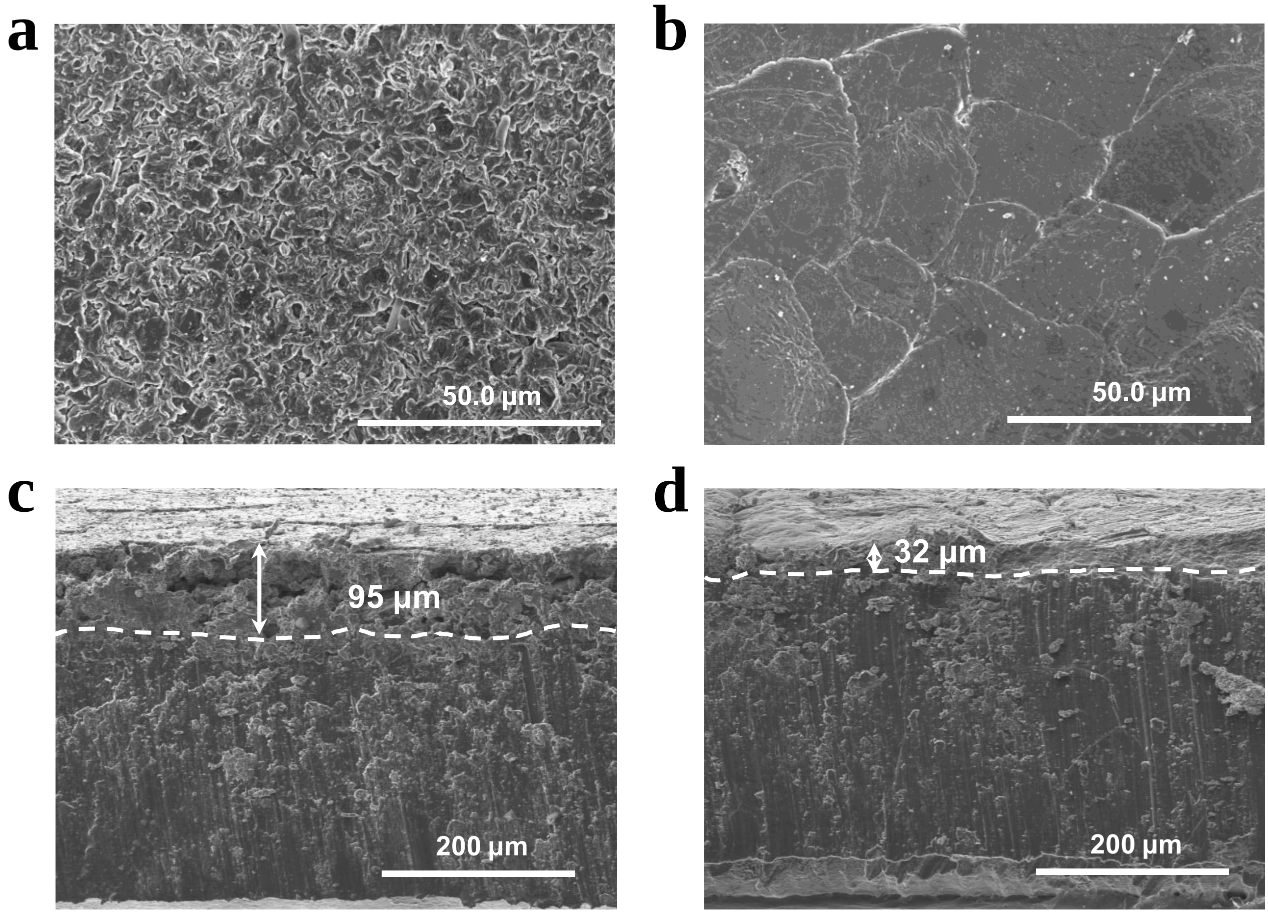

Supplementary Figure 8 Ex situ SEM images of the **(a, b)** surface morphology and (**c, d)** cross-section views of the LMA retrieved from Li||NCM811 cells using (**a, c)** BE and **(b, d)** 0.6 wt% LiHMDS after 100 cycles at 180 mA g^{-1} under 60 °C. The cell was disassembled at fully discharged state.

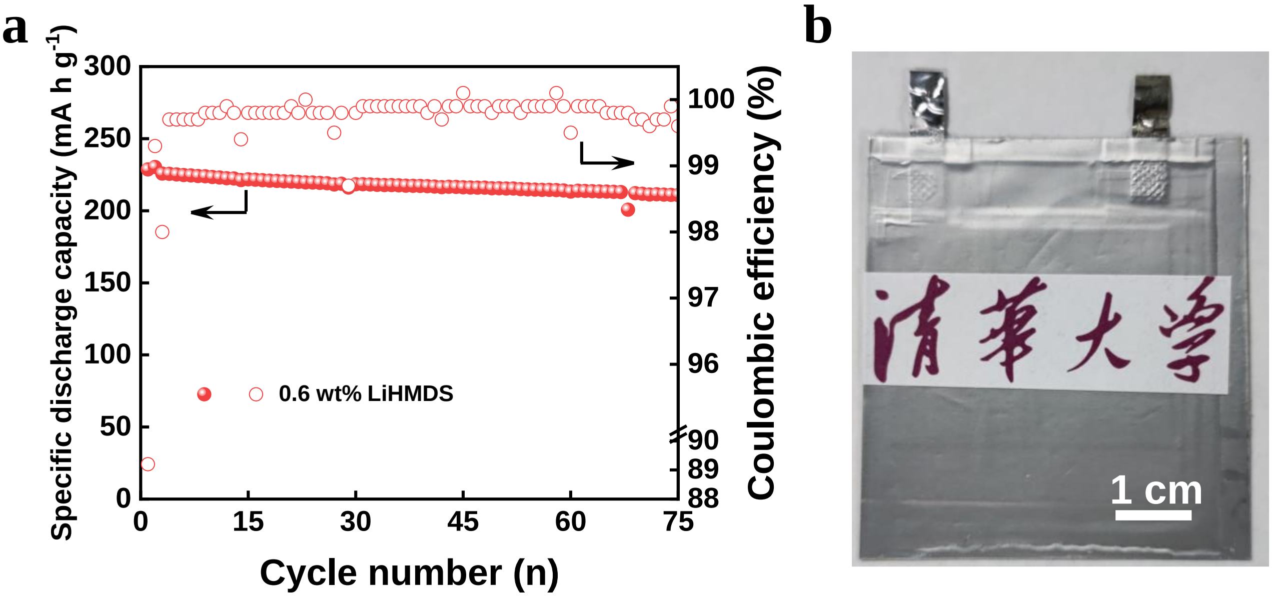

Supplementary Figure 9. (a) Cycling performance and optical photo of Li||NCM811 pouch cell with 0.6 wt% LiHMDS at charge and discharge specific current of 36 mA g^{-1}. The mass-loading of positive electrode is 10 mg cm^{-2}.**(b)** optical photograph of Li||NCM pouch cell.

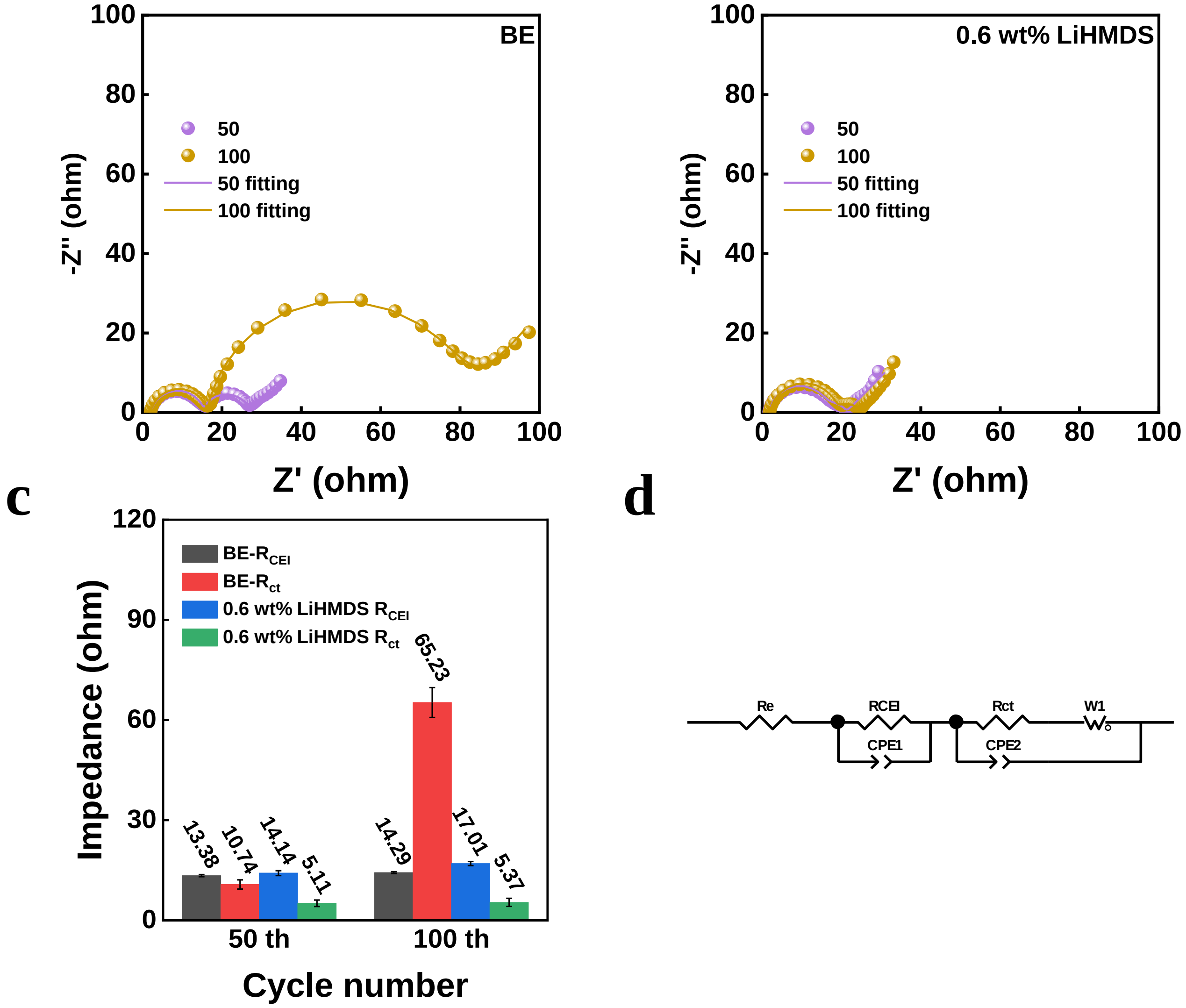

Supplementary Figure 10 Electrochemical impedance spectra of **(a)** Li||NCM811 cells and **(b)** Li||NCM811 cells with LiHMDS after different cycles at 60 °C. **(c)** R_{CEI} and R_{ct} for different cycles. The error bars represent the difference between the raw and the fit data **(d)** the equivalent circuit**.** Re means resistance of electrolyte, R_{CEI} means resistance of CEI, R_{ct} means resistance of charge transfer, CPE is constant phase angle element, W_1 is the Warburg diffusion element. The program used to fit the raw EIS data is (R_s (CPE-R_p) and R_s W_o) in the software (Zview).

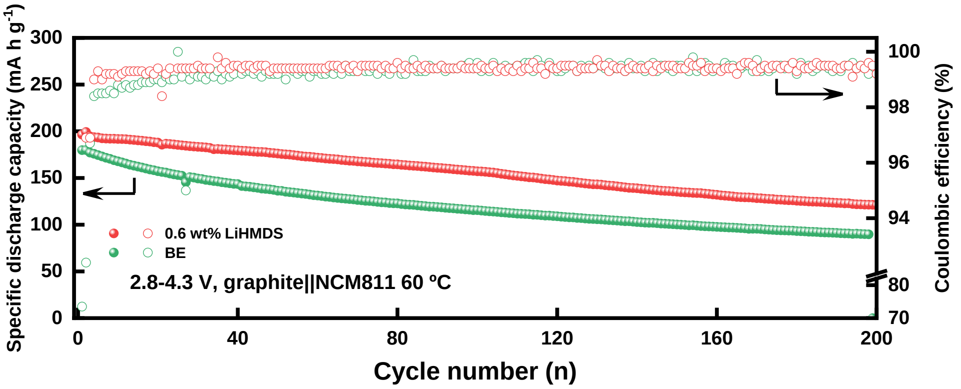

Supplementary Figure 11 Cycling performance of graphite||NCM811 full cells at 180 mA g^{-1} under 60 °C between 2.8 V and 4.3 V. The mass loading of positive electrode is 10 mg cm^{-2}.

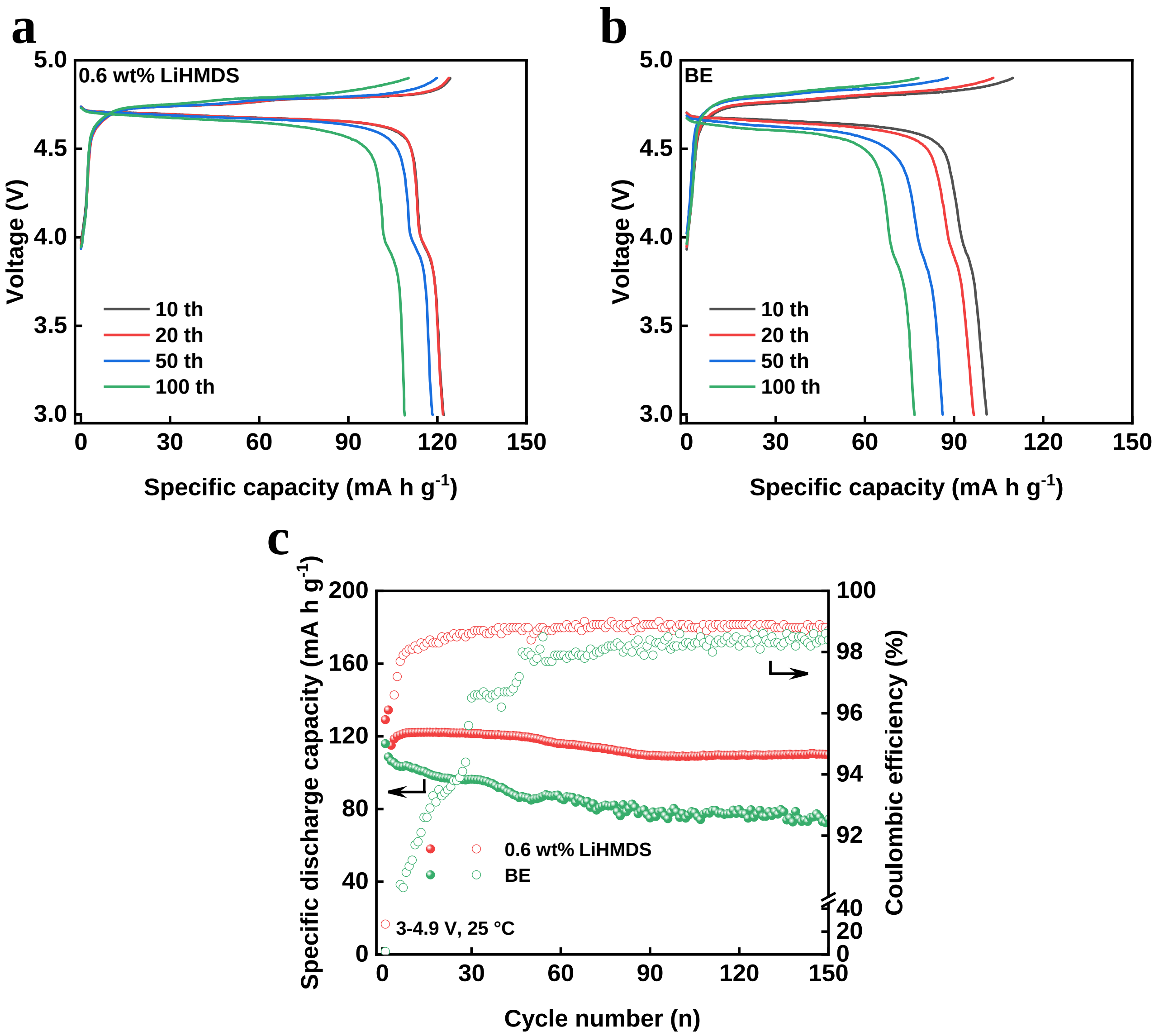

Supplementary Figure 12. Charging and discharging curves of Li||LNMO with (a) 0.6 wt% LiHMDS (b) BE, (c) Cycling performance of Li||LMNO cells at 147 mA g^{-1} under 25 ºC. The mass loading of positive electrode is 2 mg cm^{-2}.

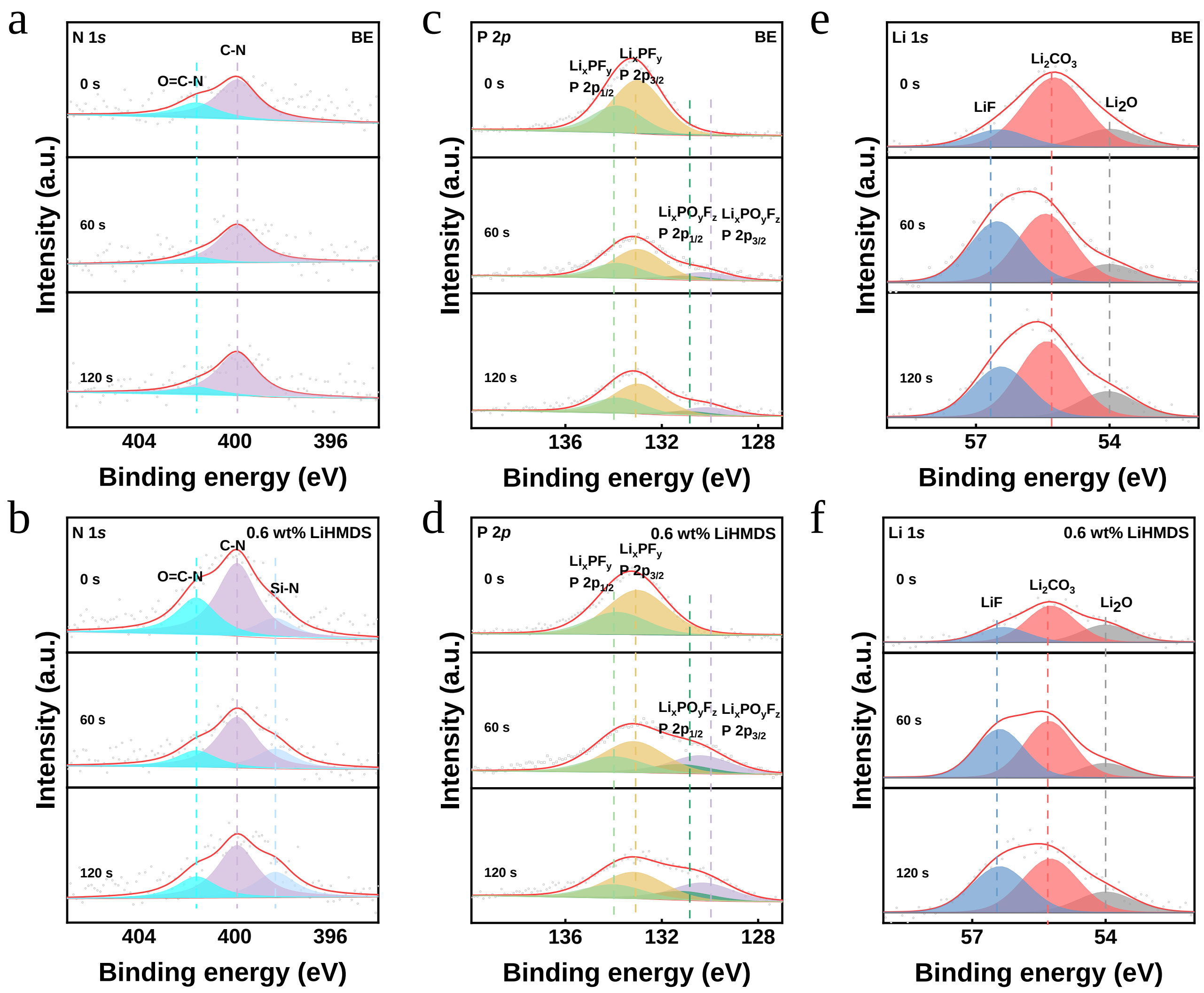

Supplementary Figure 13 Ex situ XPS measurements and analysis of NCM811 cathode retrieved from (**a, c, e**) Li||NCM811 cells and **(b, d, f)** Li||NCM811 cells with LiHMDS after 100 cycles at 180 mA g^{-1} under 60 °C. (**a, b**) N 1*s*, (**c, d**) P 2*p*, (**e, f**) Li 1*s*. The cell was disassembled at fully discharged state..

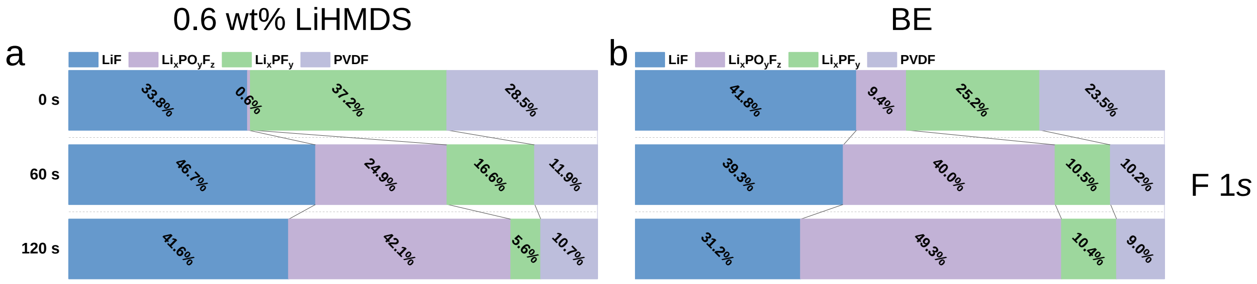

Supplementary Figure 14 Composition and distribution of different constituents of CEI on NCM811 after 100 cycles at 180 mA g^{-1} under 60 ºC using (**a**) LiHMDS and (**b)** BE obtained from XPS of (**a, b)** F 1*s*. The cell was disassembled at fully discharged state.

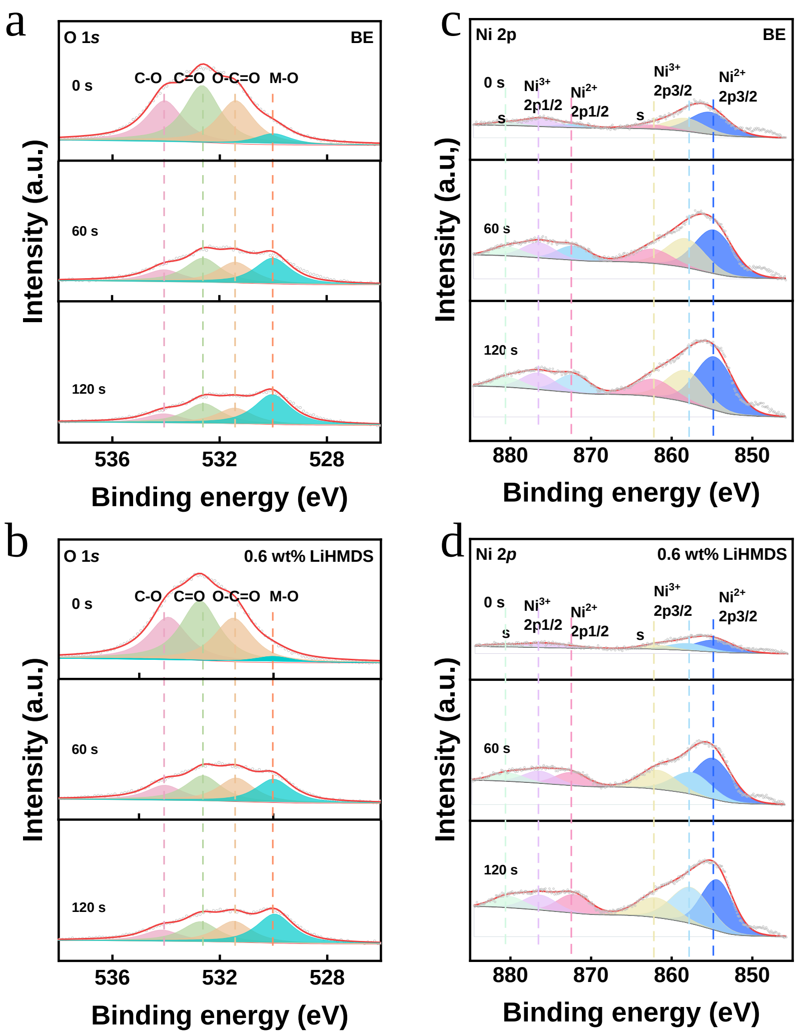

Supplementary Figure 15 Ex situ XPS measurements and analysis of NCM cathode retrieved from Li||NCM811 cells (**a, c**) and Li||NCM811 cells with LiHMDS (**b, d**) after 100 cycles at 180 mA g^{-1} under 60 °C, (**a, b**) O 1*s*, (**c, d**) Ni 2*p*. The cell was disassembled at fully discharged state.

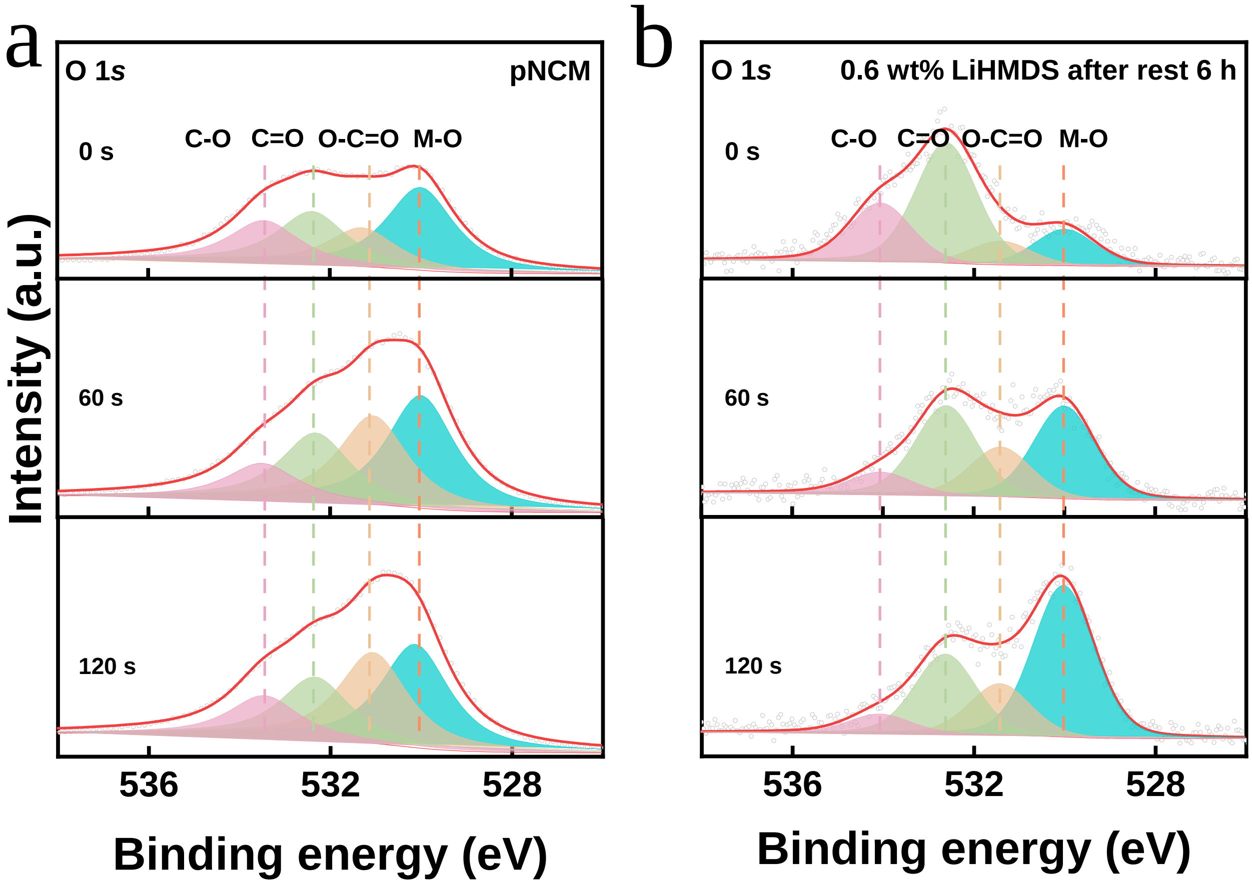

Supplementary Figure 16 Ex situ XPS measurements and analysis of **(a)** NCM particles and **(b)** NCM cathode retrieved from Li||NCM811 cells with LiHMDS rest under 60 ºC for 6 h at OCV without charging/discharging, **(a, b)** O 1*s*.

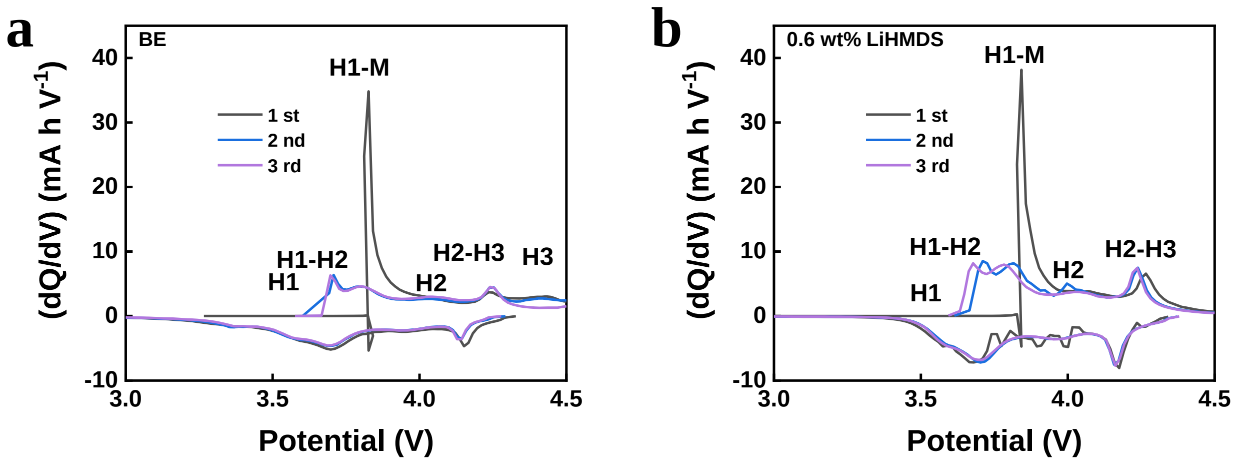

Supplementary Figure 17 Differential capacity plots for first three cycles of the NCM cathode with **(a)** BE and **(b)** LiHMDS cycled at 180 mA g^{-1} under 60 °C

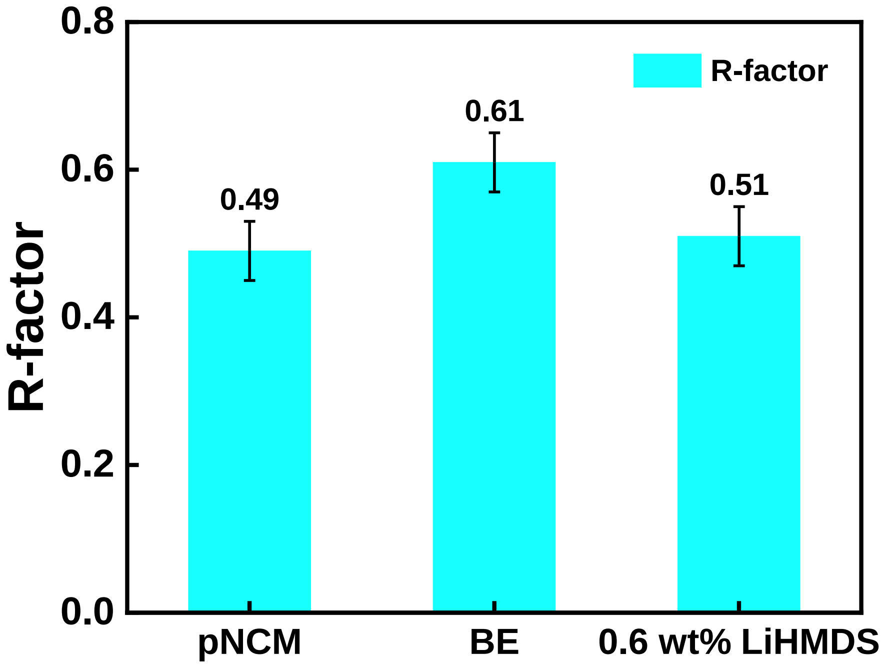

Supplementary Figure 18 Lattice parameters of pristine NCM811 cathodes and NCM811 cathodes cycling in BE and LiHMDS after 100 cycles at 180 mA g^{-1} under 60 °C. The error bar is the standard deviation of three samples. The cell was disassembled at fully discharged state.

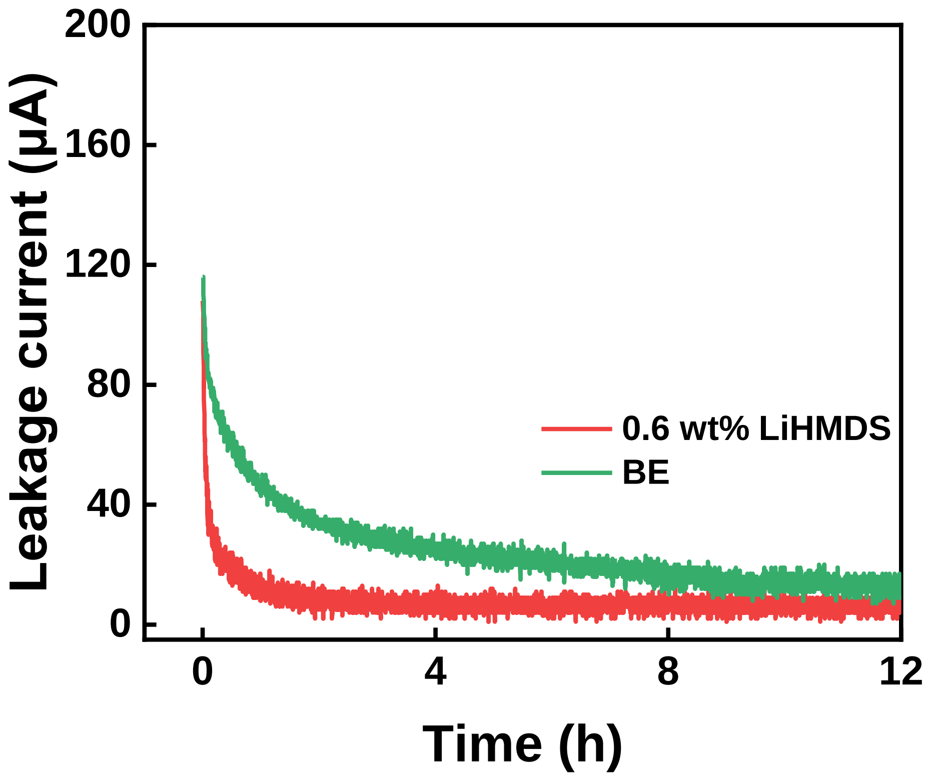

Supplementary Figure 19 Leakage currents during 4.5 V constant-voltage floating test of NCM cathodes cycled at 60 °C. The floating rest was done after Li||NCM811 coin cell assembly without cycling.

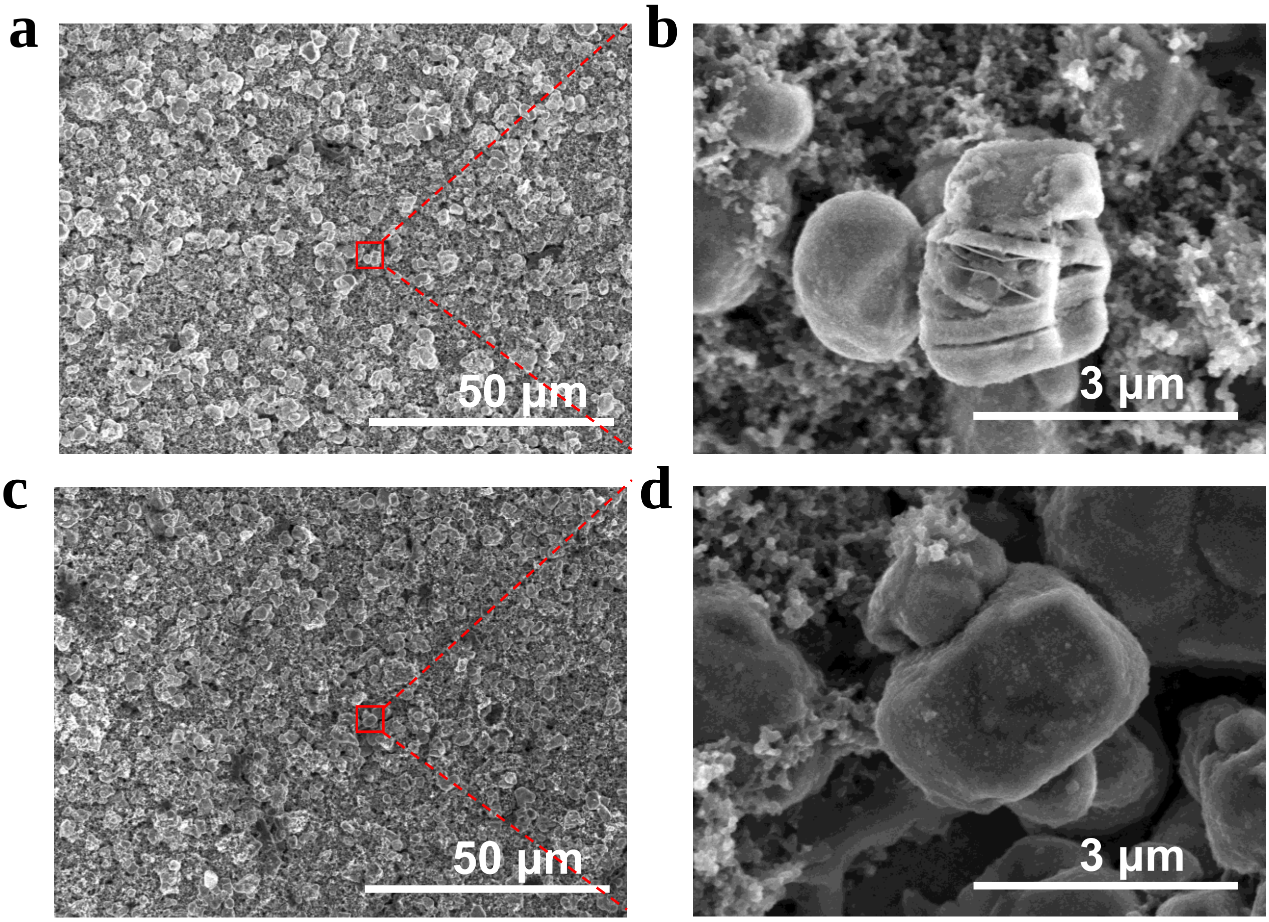

Supplementary Figure 20 Ex situ SEM characterization of NCM cathode retrieved from (**a, b**) Li||NCM811 cells and (**c, d**) Li||NCM811 cells with LiHMDS after 100 cycles at 180 mA g^{-1} under 60 °C. The cell was disassembled at fully discharged state.

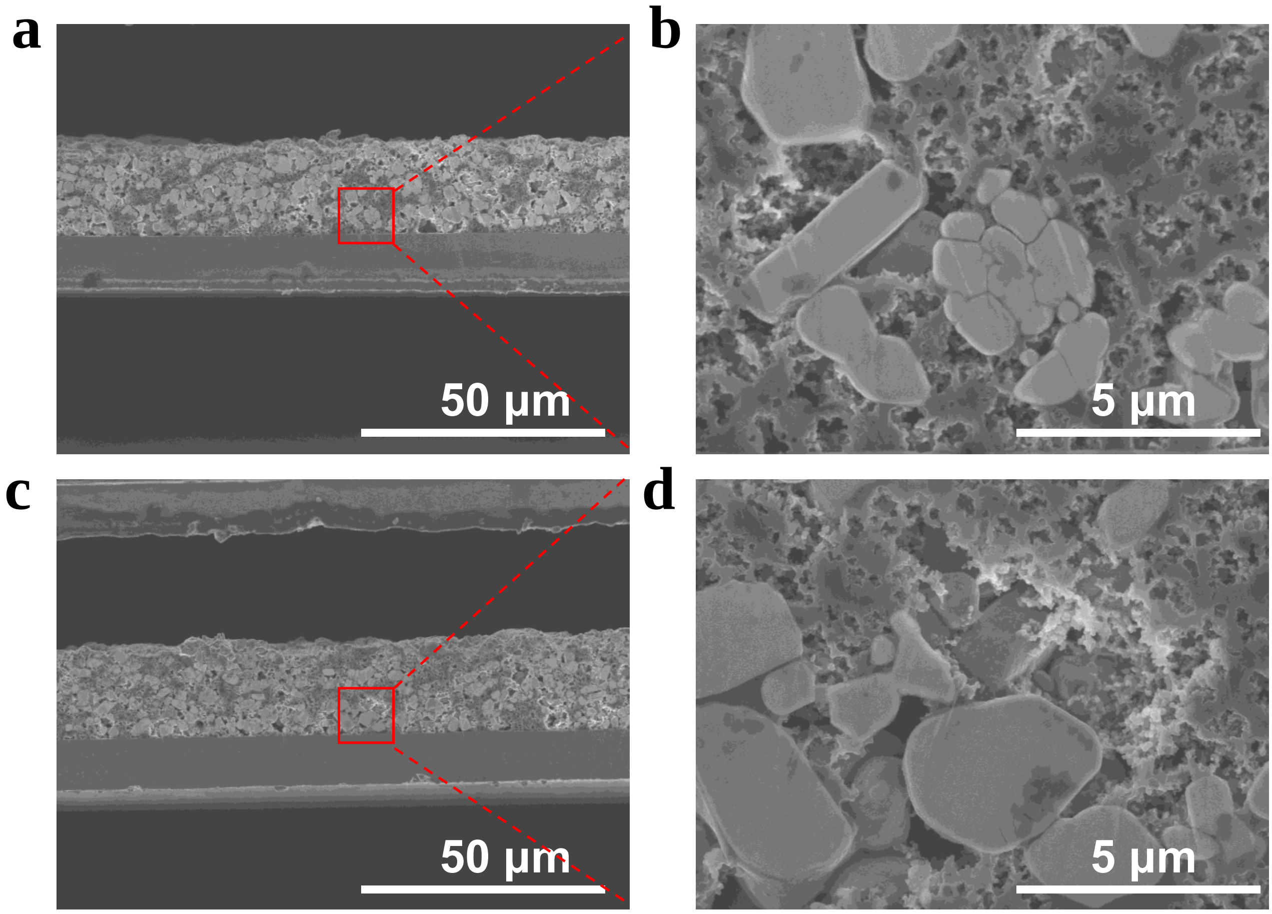

Supplementary Figure 21 cross section images of NCM cathode retrieved from (**a, b)** Li||NCM811 cells and (**c, d)** Li||NCM811 cells with LiHMDS after 100 cycles at 180 mA g^{-1} under 60 °C. The cell was disassembled at fully discharged state.

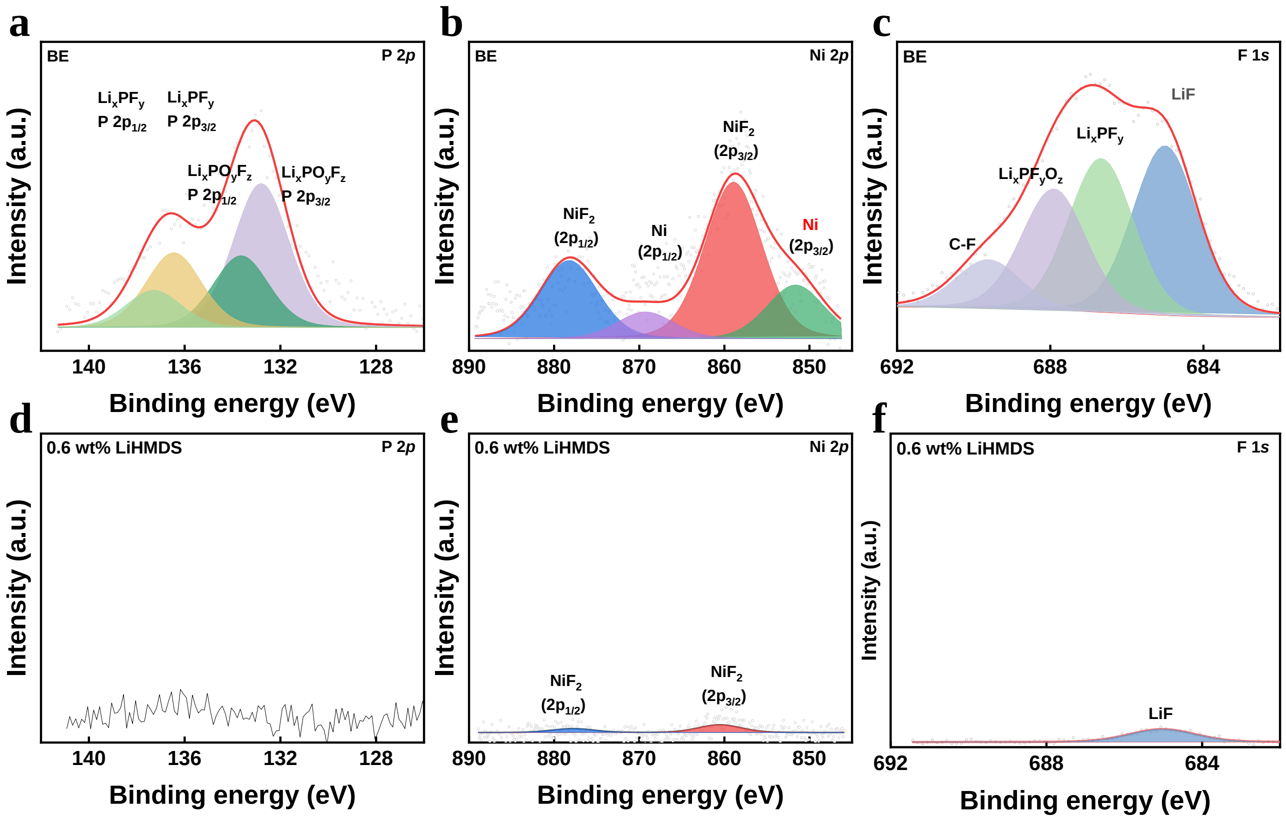

Supplementary Figure 22 Ex situ XPS measurements and analysis of lithium metal anode retrieved from (**a-c**) Li||NCM811 cells and (**d-f**) Li||NCM811 cells with LiHMDS after 100 cycles at 60 °C, (**a, d)** P 2*p*, (**b, e)** Ni 2*p***, (c, f)** F 1*s*. The cell was disassembled at fully discharged state.

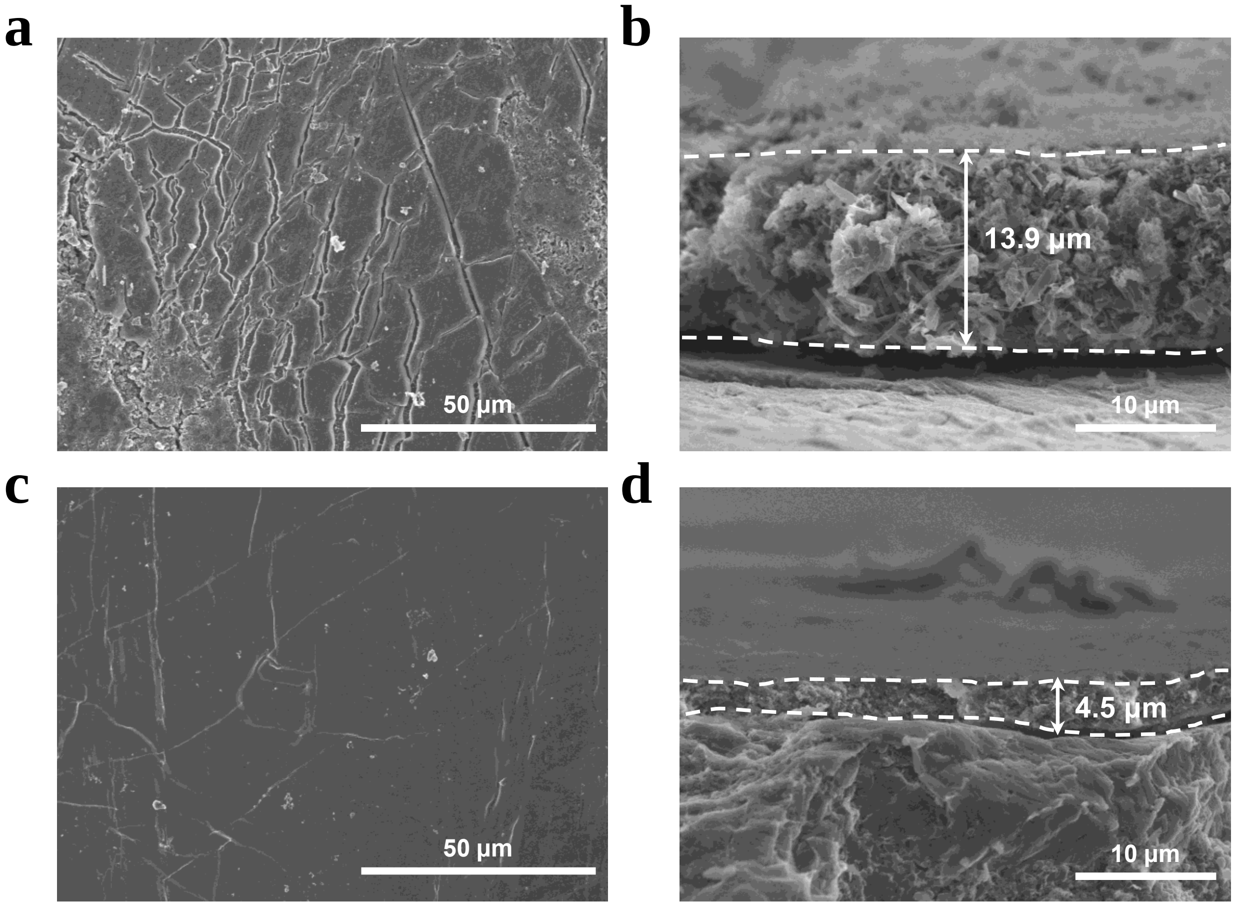

Supplementary Figure 23. Ex situ SEM characterization of the (**a, c**) surface morphology and (**b, d**) cross-section views of the LMA retrieved from the Li||NCM811 cells with (**a, b**) BE and (**c, d**) 0.6 wt% LiHMDS after 10 cycles at 180 mA g^{-1} under 60 °C. The cell was disassembled at fully discharged state.

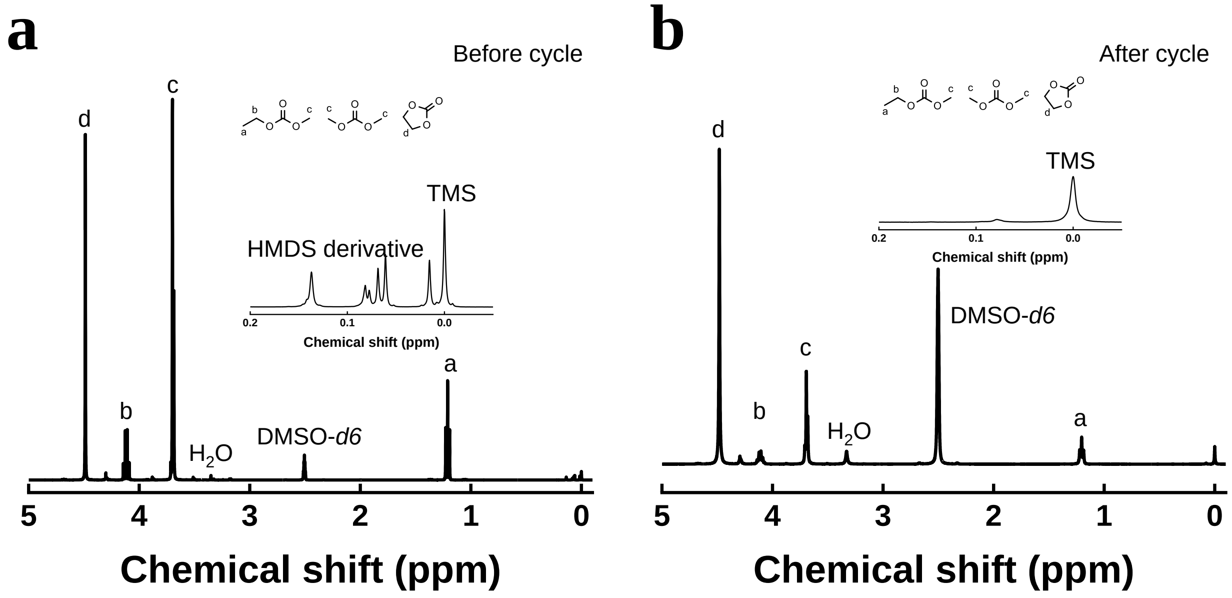

Supplementary Figure 24 Ex situ ^1H NMR measurements of LiHMDS-containing electrolyte solution sampled from Li||NCM811 cells with 0.6 wt% LiHMDS (**a**) before and (**b**) after 3 cycles at 36 mA g^{-1} under 25 ºC. The cell was disassembled at fully discharged state.

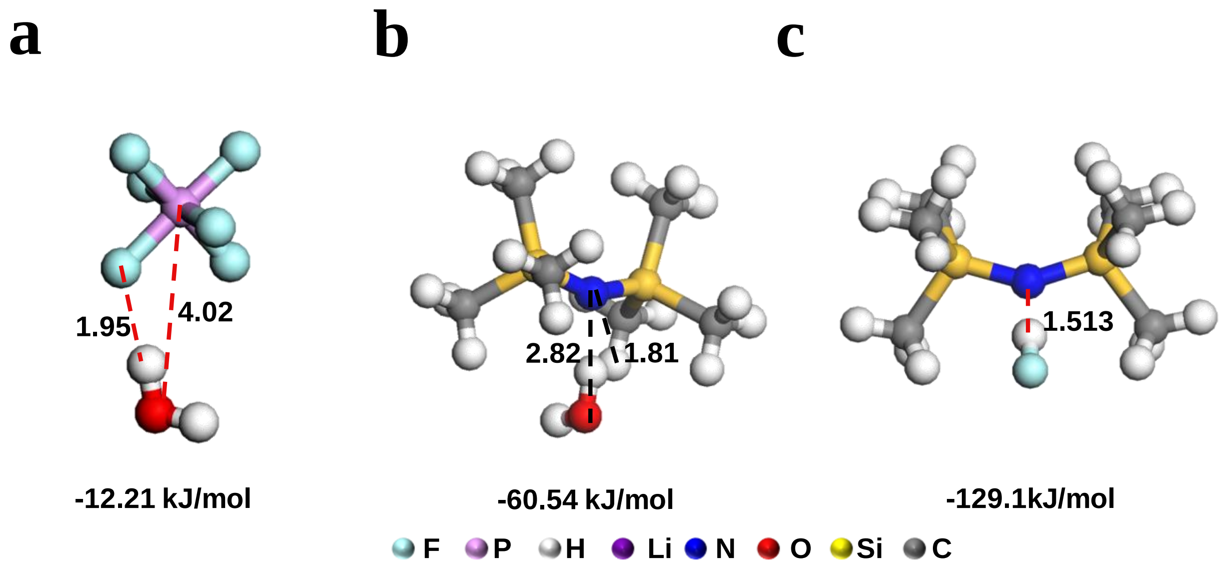

Supplementary Figure 25 Bond length and binding energy of **(a)** H_2O-PF_6^- **(b)** H_2O-HMDS$^-$ and **c** HF-HMDS$^-$. (Data with the bond length in Å).

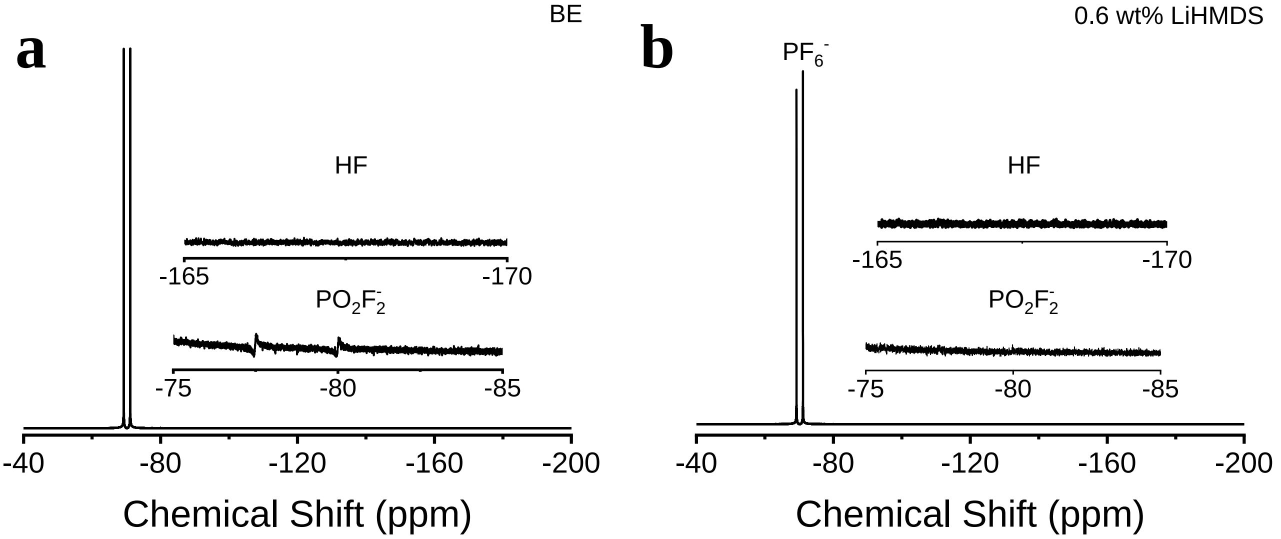

Supplementary Figure 26 Ex situ ^{19}F NMR measurements of **(a)** BE and **(b)** LiHMDS-containing electrolyte solution sampled retrieved from pristine Li||NCM811 cells rest at OCV for 7 days under 60 °C.

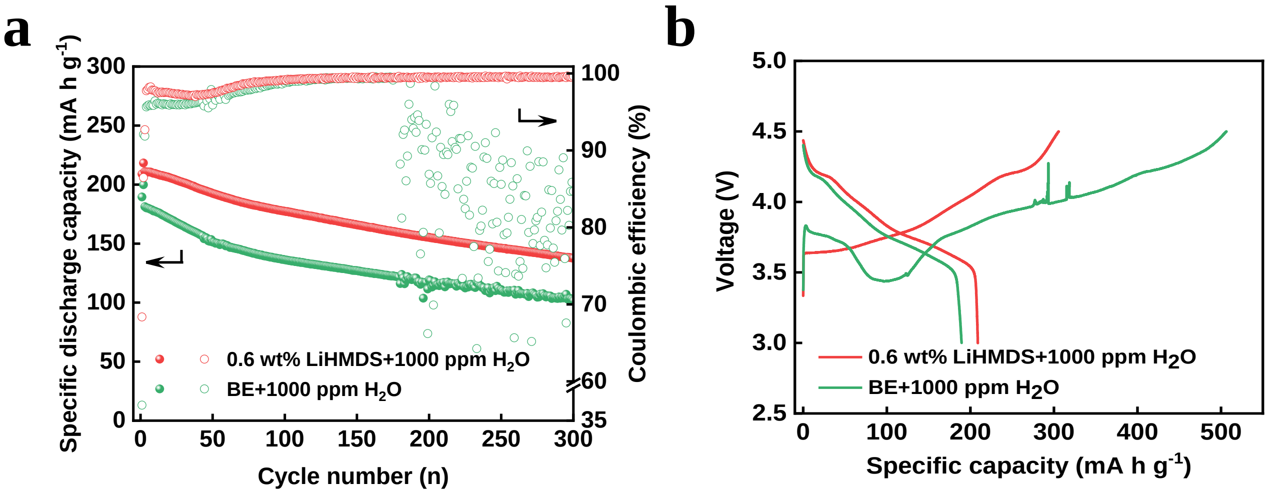

Supplementary Figure 27 (a) Cycling performance of Li||NCM811 cells cycled with 1000 ppm H_2O at 60 °C. **(b)** the first charge-discharge curve of Li||NCM811 cells at180 mA g^{-1} under 60 °C.

Supplementary Tables

Supplementary Table 1 Comparison of our work with reported electrolyte investigation works on Li||NCM batteries.

Cell configuration	Electrolyte composition	Positive electrode	Negative electrode	Operation temperature	cell voltage range	Performance	Reference
Coin cell	LiFSI-1.2DME-3TTE (75 µL)	NCM811(96 wt%, 1.5 mA h cm^{-2})	Li metal	25 ºC	2.8-4.5 V	82% (250 cycles, 66.7 mA g^{-1})	Joule 3, 1662 (2019)[1]
Coin cell	1 M $LiPF_6$ in MTFP/FEC (NM)	NCM811(80 wt%, 0.84 mA h cm^{-2})	Li metal	25 ºC	2.8-4.5 V	80% (250 cycles, 100 mA g^{-1})	ACS Energy Lett. 5, 1438 (2020)[2]
Coin cell	1.5 M $LiPF_6$ in FEC/EMC/DMC+1 wt% TMSB (80 µL)	NCM811(98wt%, 11.14 mg cm^{-2})	Li metal	25 ºC	2.7-4.5 V	70% (200 cycles 2.5 mA cm^{-2})	Angew. Chem. Int. Ed. 60, 19232 (2021)[3]
Coin cell	LiFSI-1DME-3TTE (75 µL)	NCM811(96 wt%, 1.5 mA h cm^{-2})	Li metal	25 ºC	2.8-4.4 V	N/A (300 cycles 1/3 C)	Proc. Natl. Acad. Sci. U.S.A. 117, 28603 (2020)[4]

Coin cell	1 M LiFSI in DME/TFEO (75 μL)	NCM811 (96 wt%, 1.5 mA h cm^{-2})	Li metal	25 ºC	2.8- 4.4 V	80% (300 cycles, 0.5 mA cm^{-2})	Nat. Energy 4, 796 (2019)[5]
Coin cell	1 M $LiPF_6$ in HFE/FEMC/FEC (100 μL)	NCM811(NM, 2 mA h cm^{-2})	Li metal	25 ºC	2.7-4.4 V	90% (450 cycles, 100 mA g^{-1})	Nat. Nanotechnol. 13, 715 (2018)[6]
Coin cell	4.6 M LiFSI+2.3 M LiTFSI in DME (75 μL)	NCM622(90 wt%, 1.44 mA h cm^{-2})	Li metal	25 ºC	4.4 V	88% (300 cycles, 1/3 C)	Energy Environ. Sci. 12, 780 (2019)[7]
Coin cell	1 M $LiPF_6$ in EC/DEC + 5 wt% $LiNO_3$ + 0.4 wt% $Sn(OTf)_2$ (40 μL)	NCM811(NM, 4 mA h g^{-1})	Li metal	room temperature	2.8-4.3 V	89.6% (130 cycles, 1.9 mA cm^{-2})	Adv. Mater, 32, 2001740 (2020)[8]
Coin cell	0.8 M LiTFSI + 0.2 M LiDFOB + 0.05 M $LiPF_6$ in EMC/FEC + 1 wt% adiponitrile (100 μL)	$Li[Ni_{0.73}Co_{0.10}Mn_{0.15}Al_{0.02}]O_2$ (90 wt%, 10 mg cm^{-2})	Li metal	30 ºC	2.7-4.3 V	75% (830 cycles, 1.8 mA cm^{-2})	Adv. Funct. Mater. 29, 1902496 (2019)[9]

**Coin cell**	**1 M $LiFP_6$ in EC/EMC/DMC+0.6 wt% LiHMDS (50 μL)**	**NCM811(80 wt%, 2.5 mg cm^{-2})**	**Li metal**	**25 ± 1 ºC**<br>**25 ± 1 ºC**<br>**60 ± 1 ºC**	**2.8-4.3 V**<br>**3-4.5 V**<br>**3-4.5 V**	**71.3% (1000 cycles, 90 mA g^{-1})**<br>**83.3% (500 cycles, 90 mA g^{-1})**<br>**66% (500 cycles, 180 mA g^{-1})**	**Our work**
**Pouch cell**	**1 M $LiFP_6$ in EC/EMC/DMC+0.6 wt% LiHMDS (150 μL)**	**NCM811(80 wt%, 10 mg cm^{-2}, 27 cm^2)**	**Li metal**	**60 ± 1 ºC**	**3-4.5 V**	**93.4% (75 cycles, 36 mA g^{-1})**	**Our work**

NM: not mentioned

Supplementary Table 2 Fitted EIS results of Li||NCM811 cells after different cycles

Sample	RCEI(Ω)	Error (%)	Rct(Ω)	Error (%)
BE-50	13.38	2.47	10.74	12.64
BE-100	14.29	1.99	65.23	6.88
0.6 wt% LiHMDS-50	14.14	5.22	5.11	19.22
0.6 wt% LiHMDS-100	17.01	3.667	5.37	22.614

Supplementary references

1. Ren, X. et al. Enabling high-voltage lithium-metal batteries under practical conditions. *Joule* **3**, 1662-1676 (2019).
2. Holoubek, J. et al. An all-fluorinated ester electrolyte for stable high-voltage Li metal batteries capable of ultra-low-temperature operation. *ACS Energy Lett.* **5**, 1438-1447 (2020).
3. Huang, K. et al. Regulation of SEI formation by anion receptors to achieve ultra-stable lithium-metal batteries. *Angew. Chem. Int. Ed.* **60**, 19232-19240 (2021).
4. Ren, X. et al. Role of inner solvation sheath within salt-solvent complexes in tailoring electrode/electrolyte interphases for lithium metal batteries. *Proc. Natl. Acad. Sci. U.S.A.* **117**, 28603-28613 (2020).
5. Cao, X. et al. Monolithic solid–electrolyte interphases formed in fluorinated orthoformate-based electrolytes minimize Li depletion and pulverization. *Nat. Energy* **4**, 796-805 (2019).
6. Fan, X. et al. Non-flammable electrolyte enables Li-metal batteries with aggressive cathode chemistries. *Nat Nanotechnol* **13**, 715-722 (2018).
7. Alvarado, J. et al. Bisalt ether electrolytes: a pathway towards lithium metal batteries with Ni-rich cathodes. *Energy Environ. Sci.* **12**, 780-794 (2019).
8. Zhang, W. et al. Colossal granular lithium deposits enabled by the grain-coarsening effect for high-efficiency lithium metal full batteries. *Adv. Mater.* **32**, 2001740 (2020).
9. Lee, S.H., Hwang, J.-Y., Park, S.-J., Park, G.-T. & Sun, Y.-K. Adiponitrile (C6H8N2): A new bi-functional additive for high-performance Li-metal batteries. *Adv. Funct. Mater.* **29**, 1902496 (2019).
