# Supplementary material for: Lithium hexamethyldisilazide as electrolyte additive for efficient cycling of high-voltage non-aqueous lithium metal batteries
Source: Nat Commun. 2022 Nov 15;13:6966. doi: 10.1038/s41467-022-34717-4 (PMC9666536; doi:10.1038/s41467-022-34717-4)
Supplement: Supplementary file 2 — Reporting Summary [file 41467_2022_34717_MOESM2_ESM.pdf]

nature portfolio

Corresponding author(s): Yan-Bing He
Last updated by author(s): Jul 26, 2022

Reporting Summary

Nature Portfolio wishes to improve the reproducibility of the work that we publish. This form provides structure for consistency and transparency in reporting. For further information on Nature Portfolio policies, see our Editorial Policies and the Editorial Policy Checklist.

Statistics

For all statistical analyses, confirm that the following items are present in the figure legend, table legend, main text, or Methods section.

n/a	Confirmed	
☐	☒	The exact sample size (*n*) for each experimental group/condition, given as a discrete number and unit of measurement
☐	☒	A statement on whether measurements were taken from distinct samples or whether the same sample was measured repeatedly
☐	☒	The statistical test(s) used AND whether they are one- or two-sided<br>*Only common tests should be described solely by name; describe more complex techniques in the Methods section.*
☒	☐	A description of all covariates tested
☐	☒	A description of any assumptions or corrections, such as tests of normality and adjustment for multiple comparisons
☒	☐	A full description of the statistical parameters including central tendency (e.g. means) or other basic estimates (e.g. regression coefficient) AND variation (e.g. standard deviation) or associated estimates of uncertainty (e.g. confidence intervals)
☒	☐	For null hypothesis testing, the test statistic (e.g. *F*, *t*, *r*) with confidence intervals, effect sizes, degrees of freedom and *P* value noted<br>*Give P values as exact values whenever suitable.*
☒	☐	For Bayesian analysis, information on the choice of priors and Markov chain Monte Carlo settings
☒	☐	For hierarchical and complex designs, identification of the appropriate level for tests and full reporting of outcomes
☒	☐	Estimates of effect sizes (e.g. Cohen's *d*, Pearson's *r*), indicating how they were calculated

Our web collection on statistics for biologists contains articles on many of the points above.

Software and code

Policy information about availability of computer code

Data collection	No software was used to collect data.
Data analysis	Microsoft Office (version 2206, Build 15330.20264), OriginPro (2921, 9.8.0.200), MestReNova (v11.0.4-18998), LANDdt (V7.4), EC-Lab software (V11.10). MultiPak (version 9.9.0.8), ChemDraw (version 20.1.1.125). DigitalMicrograph (version 3.51.3719.0). EIS

For manuscripts utilizing custom algorithms or software that are central to the research but not yet described in published literature, software must be made available to editors and reviewers. We strongly encourage code deposition in a community repository (e.g. GitHub). See the Nature Portfolio guidelines for submitting code & software for further information.

Data

Policy information about availability of data

All manuscripts must include a data availability statement. This statement should provide the following information, where applicable:

- Accession codes, unique identifiers, or web links for publicly available datasets
- A description of any restrictions on data availability
- For clinical datasets or third party data, please ensure that the statement adheres to our policy

The data that support the findings of this study are available from the corresponding author upon reasonable request.

Human research participants

Policy information about studies involving human research participants and Sex and Gender in Research.

Reporting on sex and gender	None
Population characteristics	None
Recruitment	None
Ethics oversight	None

Note that full information on the approval of the study protocol must also be provided in the manuscript.

Field-specific reporting

Please select the one below that is the best fit for your research. If you are not sure, read the appropriate sections before making your selection.

☐ Life sciences ☐ Behavioural & social sciences ☒ Ecological, evolutionary & environmental sciences

For a reference copy of the document with all sections, see nature.com/documents/nr-reporting-summary-flat.pdf

Ecological, evolutionary & environmental sciences study design

All studies must disclose on these points even when the disclosure is negative.

Study description	The lithium hexamethyldisilazide (LiHMDS) additive enables stable cycling of Li||NCM811 batteries with a cut-off voltage up to 4.5 V and an operation temperature up to 60 °C. The LiHMDS completely eliminates the HF and H2O in electrolyte, preferentially constructs a thin and robust cathode electrolyte interface (CEI) before oxidation of electrolyte. This CEI effectively prevents the Ni dissolution of NCM811 to mitigate the irreversible phase transformation from layered structure to rock-salt phase and suppress the side reactions with electrolyte. The trace LiHMDS endows the Li||NCM811 batteries stably cycling 1000 times at 25 °C and 500 times at 60 °C under 4.5 V.
Research sample	LiPF6 based electrolyte with/without LiHMDS additive in Li||NCM811 cells.
Sampling strategy	Sampling was not used in this study.
Data collection	The data was collected by equipments automatically
Timing and spatial scale	No timing and spatial scale limitation.
Data exclusions	All the data was used without exclusion.
Reproducibility	The results can be well repeated.
Randomization	Randomization was not used in this study.
Blinding	Investigators were not blinded in this study as quantitative measures were used to measure our results.

Did the study involve field work? ☐ Yes ☒ No

Reporting for specific materials, systems and methods

We require information from authors about some types of materials, experimental systems and methods used in many studies. Here, indicate whether each material, system or method listed is relevant to your study. If you are not sure if a list item applies to your research, read the appropriate section before selecting a response.

Materials & experimental systems

n/a	Involved in the study
☒	☐ Antibodies
☒	☐ Eukaryotic cell lines
☒	☐ Palaeontology and archaeology
☒	☐ Animals and other organisms
☒	☐ Clinical data
☒	☐ Dual use research of concern

Methods

n/a	Involved in the study
☒	☐ ChIP-seq
☒	☐ Flow cytometry
☒	☐ MRI-based neuroimaging
